# Synthesis and Characterization of a Copper Complex Supported by a Z‑type Sb^V^ Ligand: XPS and DFT Study of Electronic Structure

**DOI:** 10.1021/acs.organomet.5c00420

**Published:** 2026-02-05

**Authors:** Christopher K. Webber, Macarena G. Alférez, Farzad Bastani, Jugal Kumawat, Fanji Kong, Zoë M. Gehman, Xinrui Ou, Diane A. Dickie, Daniel H. Ess, Petra Reinke, T. Brent Gunnoe

**Affiliations:** † Department of Chemistry, 2358 University of Virginia, Charlottesville, Virginia 22904, United States; ‡ Department of Materials Science and Engineering, University of Virginia, Charlottesville, Virginia 22904, United States; § Department of Chemistry and Biochemistry, 6756 Brigham Young University, Provo, Utah 84604, United States

## Abstract

We describe the synthesis and characterization of a Cu­(I) complex, {Q_3_Sb­(*o*-chlor)}­Cu­(OTf) (Q = 8-quinolinyl; OTf = trifluoromethanesulfonate; *o*-chlor = *o*-choranil), supported by the Sb­(V) ligand Q_3_Sb­(*o*-chlor). The complex {Q_3_Sb­(*o*-chlor)}­Cu­(OTf) was experimentally characterized via ^1^H, ^13^C­{^1^H}, and ^19^F­{^1^H} NMR spectroscopy, elemental analysis, single-crystal X-ray diffraction, and X-ray photoelectron spectroscopy (XPS) as well as examined computationally with density functional theory (DFT) calculations. Variable temperature ^1^H NMR spectroscopy (20 to −110 °C) indicates temperature-dependent fluxional processes for {Q_3_Sb­(*o*-chlor)}­Cu­(OTf) and uncoordinated Q_3_Sb­(*o*-chlor). The electron density of Cu for {Q_3_Sb­(*o*-chlor)}­Cu­(OTf) was probed by comparing Cu^II^/Cu^I^ redox potential and Cu 2p electron binding energies, using XPS, with a related non-Sb-containing complex, (TMQA)­Cu­(OTf) (TMQA = tris­(quinolin-2-ylmethyl)­amine). The *E*_1/2_ of the Cu^II^/Cu^I^ redox of {Q_3_Sb­(*o*-chlor)}­Cu­(OTf) is shifted 670 mV more positive than that of (TMQA)­Cu­(OTf). XPS spectra of {Q_3_Sb­(*o*-chlor)}­Cu­(OTf) and (TMQA)­Cu­(OTf) indicate a 0.8 eV higher Cu 2p binding energy for {Q_3_Sb­(*o*-chlor)}­Cu­(OTf). Computational studies of the molecular orbitals and localized natural bonding orbitals (NBOs) are consistent with a weak Cu­(I) → Sb­(V) interaction for {Q_3_Sb­(*o*-chlor)}­Cu­(OTf), for which Sb­(V) acts as a Z-type ligand.

## Graphical abstract

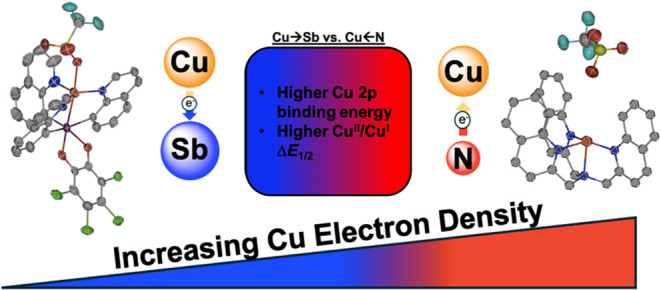

## Introduction

The Covalent Bonding Classification published by Green divides 2-center-2-electron bonding interactions into three classes: L-type, X-type, and Z-type.^1^ L- and X-type ligands, for which the ligand acts as a 2-electron or 1-electron donor, respectively, have dominated organometallic research. Z-type ligands that accept a lone pair from coordinated metals into an empty ligand orbital have received comparatively less attention. Complexes with Z-type ligands have been the focus of recent studies due to their unique electronic structures,^2–4^ and they provide an opportunity to access electronic structures that have been demonstrated to benefit catalytic processes.^5–8^

The bonding nature of Sb {i.e., functioning as either a σ-donor (L-type) or σ-acceptor (Z-type)} upon coordination to transition metals can vary depending on the Sb oxidation state.^4,9^ Our group has investigated a family of quinoline-substituted Sb ligands (Figure 1), for which the quinoline functions in a bridging mode, to prepare Pt–Sb complexes that form various redox and coordination isomers.^10,11^ Further, the study of Pt­(II)–Sb­(III) complexes with acetate ligands on Pt demonstrated an increase in nucleophilic reactivity with alkyl chlorides compared to similar Pt-acetate complexes without a Pt–Sb group. A similar Rh–Sb complex was recently reported.^12^ Further work with this family of Sb ligands demonstrated that an Sb­(V) pro-ligand, Q_2_SbPh­(*o*-chlor) (Q = 8-quinolinyl; *o*-chlor = *o*-chloranil), reacts with low valent Ir­(I) to form an Ir­(II)–Sb­(IV) complex.^13^

**Figure 1:**
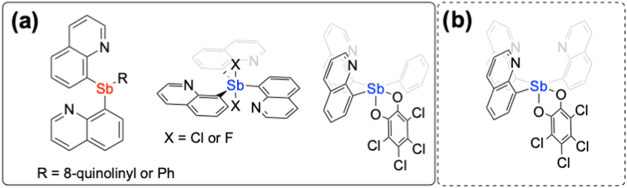
(a) Previously reported Sb ligands differing in oxidation state {red = Sb­(III), blue = Sb­(V)}. (b) Sb­(V) ligand studied in this report.

First-row transition metal Z-type complexes are relatively less explored than the second row and third row. Boron-based Z-type ligands coordinated to Ni and Fe have been reported.^14,15^ Similarly, Cu­(I) → B boratrane complexes have been reported using thiopyridazine and phosphine bridging motifs (Figure 2a).^16,17^ Cu­(I) cluster compounds supported by Sb­(III) have been isolated, some of which have been used for catalytic nitrene transfer.^18–20^ More recently, a mononuclear Cu­(I)–Sb­(III) complex was reported with a bonding interaction between Cu and Sb (Figure 2b).^21^

**Figure 2:**
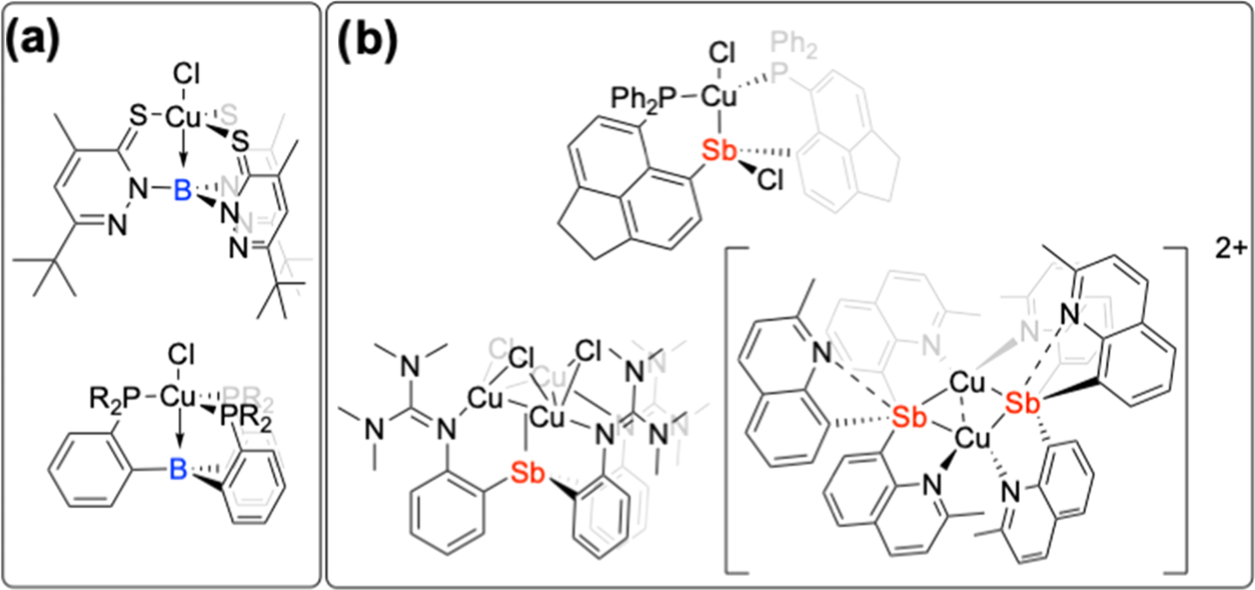
(a) Previous examples of Cu complexes containing a Cu → B interaction.^16,17^ (b) Recent examples of Cu–Sb complexes.^18–21^

Although some groups have isolated Cu complexes with Sb, to our knowledge, there have been no isolated examples of a Cu­(I) center with a Z-type interaction with Sb­(V). Therefore, we sought to isolate a mononuclear Cu­(I)–Sb­(V) complex to study the electron-withdrawing properties of a Z-type Sb­(V) coordinated to Cu­(I). Herein, we describe the isolation and characterization of a Cu–Sb complex by measuring the electron density on Cu via cyclic voltammetry and X-ray Photoelectron Spectroscopy (XPS). Also, density functional theory (DFT) calculations were used to examine the bonding interaction between Cu and Sb and compare this with a complex containing a Cu ← N interaction.

## Results and Discussion

Building on previous work with quinoline-substituted Sb ligands,^10,12,13,19,22^ we sought to study the influence of bonding to Sb­(V) on a molecular Cu complex. Previously, we found that stirring the Sb­(III) pro-ligand tri­(quinolin-8-yl)­stibane (Q_3_Sb) with 2-electron oxidants such as dichloro­(phenyl)-λ^3^-iodane or difluoro­(phenyl)-λ^3^-iodane allows the isolation of Sb­(V) compounds that can be coordinated to Rh­(I) precursors.^22^ More recently, we found that the pro-ligand 8,8′-(phenylstibanediyl)­diquinoline (Q_2_SbPh) reacts with 3,4,5,6-tetrachlorocyclohexa-3,5-diene-1,2-dione (*o*-chlor) to give Q_2_SbPh­(*o*-chlor), which coordinates low valent Ir­(I).^13^ In order to further explore these Sb­(V) ligands, we reacted Q_3_Sb (**1**) with *o*-chlor in dichloromethane (DCM) under an inert atmosphere to give the Sb­(V) pro-ligand Q_3_Sb­(*o*-chlor) (**2**) (Figure 3a). Compound **2** was characterized by ^1^H and ^13^C­{^1^H} NMR spectroscopy, single-crystal X-ray diffraction, and elemental analysis.

**Figure 3:**
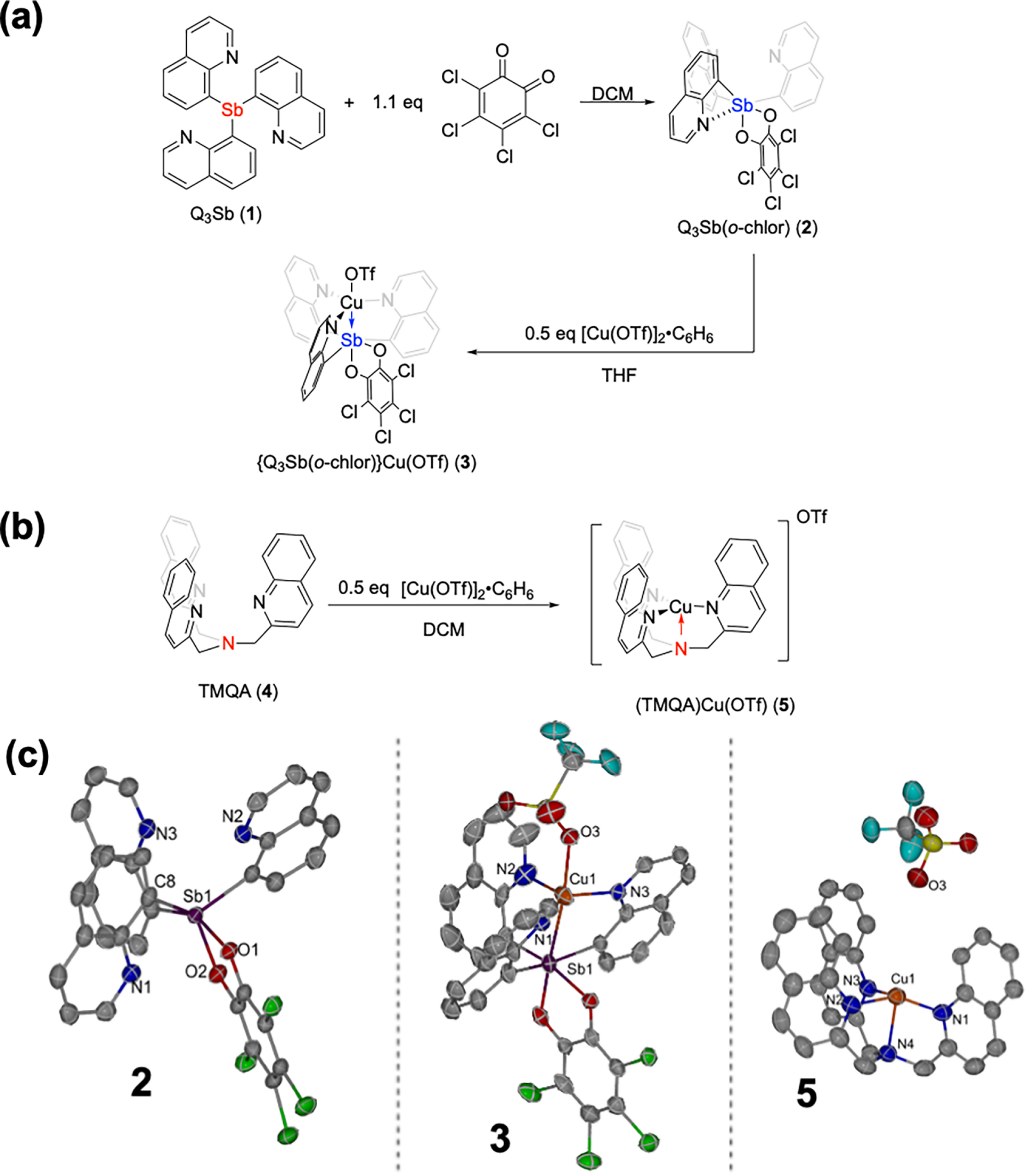
(a) Synthesis of Q_3_Sb­(*o*-chlor) (**2**) and {Q_3_Sb­(*o*-chlor)}­Cu­(OTf) (**3**) from previously reported Q_3_Sb (**1**). (b) Synthesis of (TMQA)­Cu­(OTf) (**5**). (c) Oak Ridge thermal-ellipsoid plots (ORTEPs) of Q_3_Sb­(*o*-chlor) (**2**) (left), {Q_3_Sb­(*o*-chlor)}­Cu­(OTf) (**3**) (middle), and (TMQA)­Cu­(OTf) (**5**) (Right). Ellipsoids were drawn at 50% probability. Hydrogen atoms and noncoordinating solvent are removed for clarity (right). Note: two molecules of **5** are present in the asymmetric unit of the crystal structure. Only one molecule of **5** is shown in the figure. Selected bond (and through space) lengths (Å) and angles (deg) for **2**: N1–Sb1 2.704(3), N2–Sb1 3.140(3), N3–Sb1 3.027(3), C8–Sb1 2.126(4), O1–Sb1 2.092(3), O1–Sb1–O2 78.44(10), C8–Sb1–C17 124.06(15). Selected bond lengths (Å) and angles (deg) for **3**: Cu1–Sb1 2.6761(11), Cu1–N1 1.979(5), Cu1–N2 2.226(6), Cu1–N3 2.010(5), Cu1–O3 2.198(4), N1–Cu1–N2 122.9(2), N2–Cu1–N3 99.5(2), N3–Cu1–N1 135.6(2). Selected bond (and through space) lengths (Å) and angles (deg) for **5**: Cu1–N1 1.941(5), Cu1–N2 2.110(5), Cu1–N3 1.971(5), Cu1–N4 2.209(5), Cu1–O3 5.394(5), N1–Cu1–N2 116.7(2), N2–Cu1–N3 93.5(2), N3–Cu1–N1 145.4(2), Cu2–N5 2.003(5), Cu2–N6 1.981(5), Cu2–N7 2.026(5), Cu2–N8 2.189(5), Cu2–O4 4.428(5), N5–Cu2–N6 125.29(19), N6–Cu2–N7 118.6(2), N7–Cu2–N5 110.98(19).

The solid-state structure (Figure 3c, left) indicates a close through space distance between one nitrogen and Sb compared to the other two quinoline nitrogen atoms (N1–Sb1 2.704(3) Å vs N2–Sb1 3.140(3) Å and N3–Sb1 3.027(3) Å) indicating Lewis acidity of Sb­(V) and suggesting the presence of an interaction that could be considered a pnictogen bond similar to previously reported Q_2_SbPh­(*o*-chlor).^13,23^ The ^1^H NMR spectrum of **2** showed only six resonances corresponding to quinoline, indicating that, although there is a potential pnictogen-bonding interaction with Sb, the quinolines are exchanging rapidly on the NMR time scale at room temperature.

We found that **2** reacts with [Cu­(OTf)]_2_·C_6_H_6_ to form {Q_3_Sb­(*o*-chlor)}­Cu­(OTf) (**3**) in tetrahydrofuran (THF) in 73% isolated yield under air-free conditions in a dinitrogen-filled glovebox (Figure 3a) with a trace intractable impurity (the impurity can be removed with THF with a loss in overall yield). Complex **3** was characterized by ^1^H and ^13^C­{^1^H} NMR spectroscopy, single-crystal X-ray diffraction, and elemental analysis. The solid-state structure of complex **3** (Figure 3c, middle) shows a close distance between Cu and Sb of 2.6761(11) Å, which is shorter than the sum of the covalent radii (2.71 Å) and the van der Waals radii (*r*_vdW_ = 4.85 Å).^24,25^ The ^1^H NMR and ^13^C­{^1^H} spectra of **3** at room temperature in CD_2_Cl_2_ are similar to **2** with 6 and 12 resonances, respectively, although a downfield shifted proton resonance at 9.64 ppm is observed, which, as indicated previously,^10^ is likely due to a through space interaction with the coordinated anion (in this case, triflate) and a quinoline hydrogen.

We prepared tris­(quinolin-2-ylmethyl)­amine (TMQA, **4**) as a tetradentate non-Sb-containing ligand to act as a control for ligand **2** from a previously reported method.^26^ (TMQA)­Cu­(OTf) (**5**) was prepared from a reaction of [Cu­(OTf)]_2_·C_6_H_6_ and **4** to exchange a Lewis acidic Sb­(V) bridgehead to a Lewis basic nitrogen while keeping the coordination environment similar to complex **3** (Figure 3b). The distance between Cu and the closest oxygen of the triflate anion increases significantly for **5** versus **3** (4.428(5) vs 2.198(4) Å for **5** and **3**, respectively), indicating an outer sphere triflate group for **5** but an inner sphere for **3**, and potentially indicating a more electron-deficient Cu center in complex **3** versus **5**.

### Variable Temperature NMR Studies of Q_3_Sb­(*o*-chlor) (**2**) and {Q_3_Sb­(*o*-chlor)}­Cu­(OTf) (**3**)

The solid-state structures of both **2** and **3** indicate the potential presence of mirror symmetry (complexes **2** and **3** do not contain crystallographic mirror symmetry; however, in solution, we hypothesize that the quinolines trans to one another would chemically equilibrate on the NMR time scale) that would give two chemically equivalent quinoline proton environments and one unique quinoline. This contrasts with the symmetric ^1^H NMR spectra obtained at room temperature for **2** and **3** (i.e., only six proton resonances from quinoline groups are observed). The ^1^H NMR spectra indicate that both **2** and **3** are fluxional at room temperature (Figures 4 and 5). Both **2** and **3** were subjected to variable temperature ^1^H NMR studies between room temperature and −110 °C (−110 °C was the lowest temperature accessible utilizing a 60:27:13% by volume mixture of CD_2_Cl_2_/CCl_4_/CDCl_3_ as the solvent).^27^ The spectra of **2** varied at low temperatures but did not reach slow exchange down to −110 °C, indicating a relatively low activation barrier for exchange between the quinoline groups (Figure 4b). Although we were unable to synthesize a stable Cu­(I) complex using previously synthesized Q_2_SbPh­(*o*-chlor),^13^ we performed variable temperature (to −110 °C) ^1^H NMR studies of Q_2_SbPh­(*o*-chlor) and observed a larger degree of line broadening than was observed for **2** (Figure S8).

**Figure 4:**
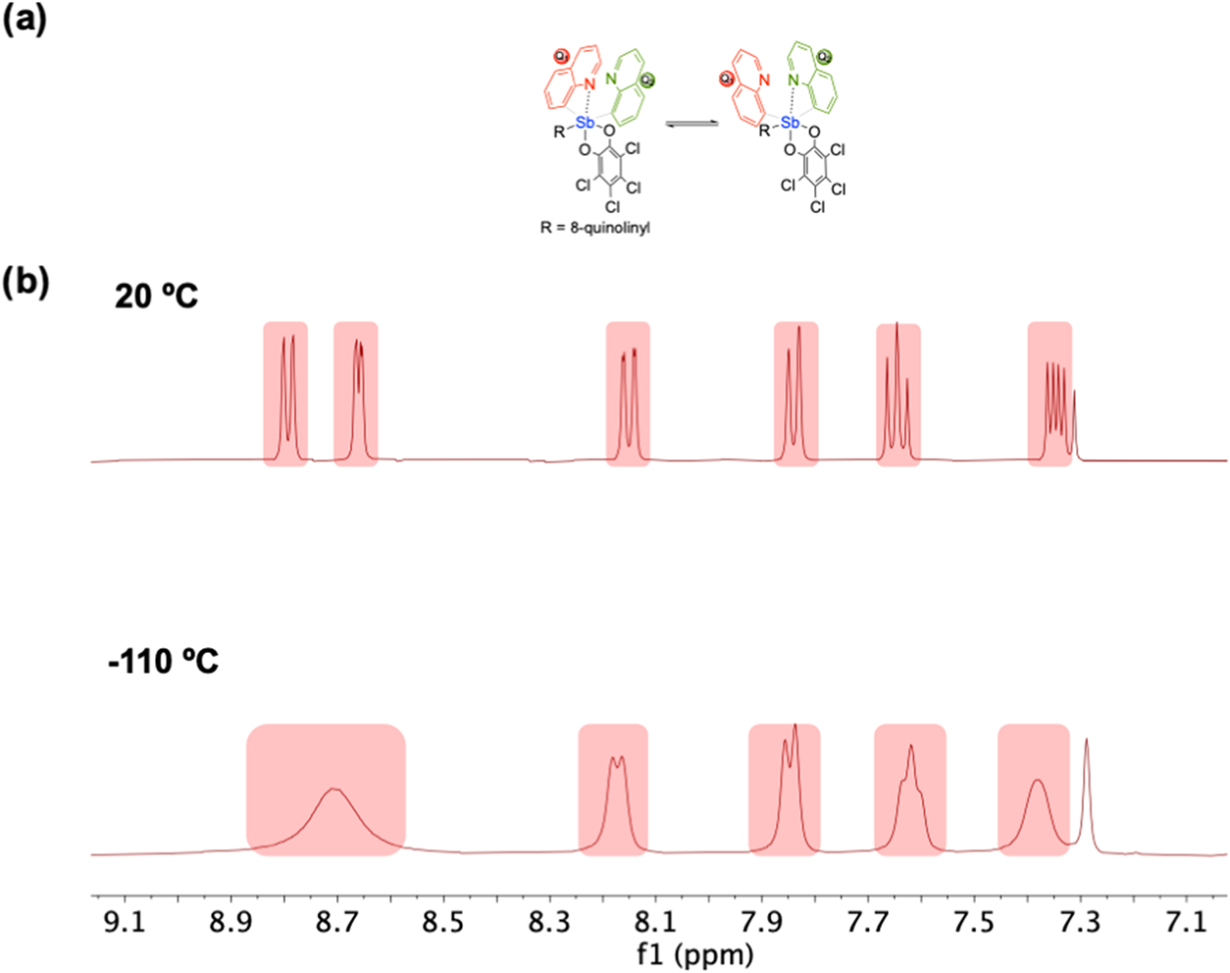
(a) Depiction of proposed fluxionality for Q_3_Sb­(*o*-chlor) (**2**) for which pnictogen-bonding interactions are exchanged between quinolines. (b) ^1^H NMR spectroscopy of **2** (60:27:13% by volume mixture of CD_2_Cl_2_/CCl_4_/CDCl_3_, 400 MHz)^27^ at 20 and −110 °C demonstrating line broadening of the peaks, indicating a likely fluxional process. See Supporting Information Figure S8 for the variable temperature ^1^H NMR spectra of Q_2_SbPh­(*o*-chlor).

**Figure 5:**
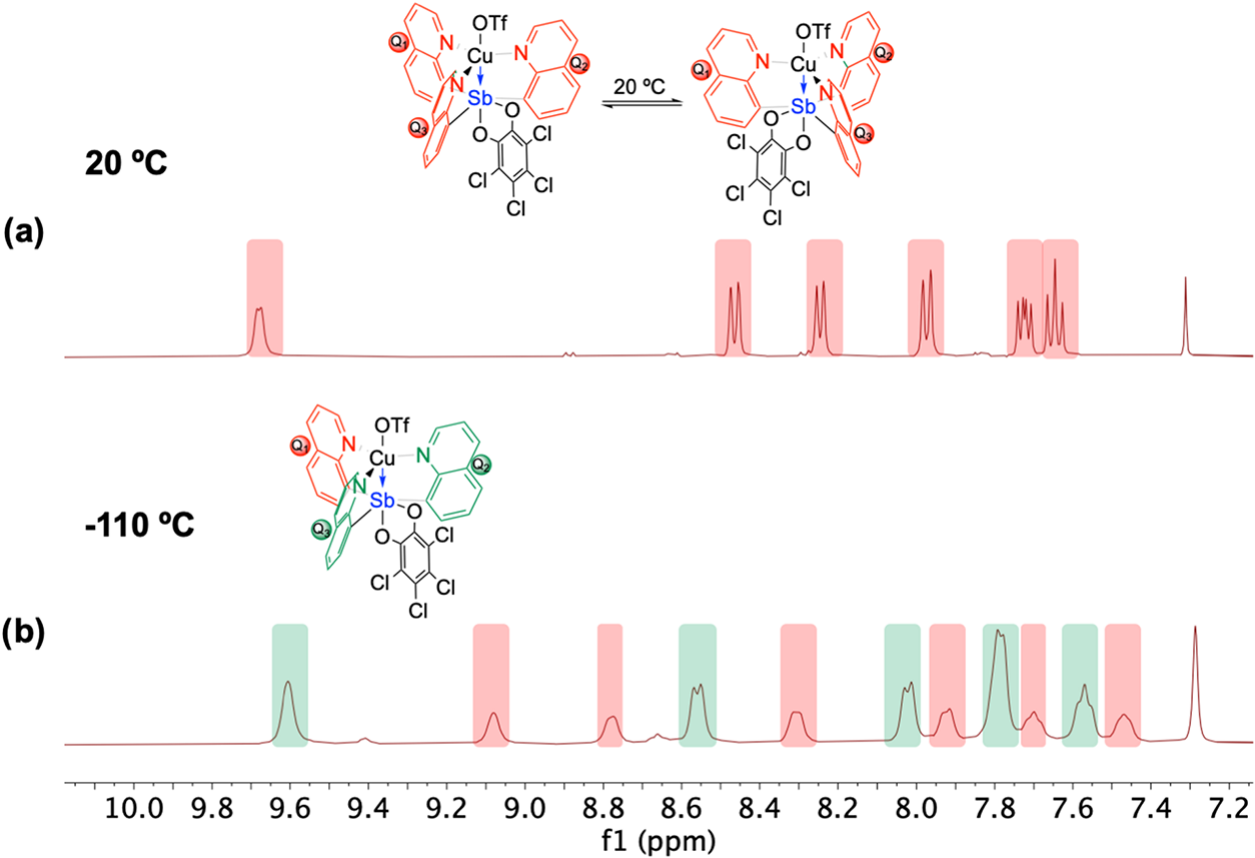
(a) ^1^H NMR spectroscopy of {Q_3_Sb­(*o*-chlor)}­Cu­(OTf) (**3**) (60:27:13% by volume mixture of CD_2_Cl_2_/CCl_4_/CDCl_3_, 400 MHz)^27^ at 20 °C and depiction of potential fluxionality at 20 °C. (b) ^1^H NMR spectroscopy of **3** (60:27:13% by volume mixture of CD_2_Cl_2_/CCl_4_/CDCl_3_, 400 MHz)^27^ at −110 °C demonstrating the decoalescence of the exchanging resonances at −110 °C into resonances that integrate for a 2:1 ratio. Note: due to overlap, the peak at 7.8 ppm integrates for four protons. There is a trace of an intractable impurity in the aromatic region.

The solid-state structure of **3** shows a potential molecular mirror plane (although not crystallographically as noted above) through one quinoline and the *o*-chloranil group, which is expected to give rise to a 2:1 ratio of quinoline protons in the ^1^H NMR spectrum, as was observed in our previous studies of Pt and Rh complexes with the same ligand.^10,22^ In contrast, the ^1^H NMR spectrum of **3** at 20 °C shows only a single set of resonances for the quinoline groups, which indicates that a fluxional process is likely exchanging the quinoline protons on the NMR time scale (Figure 5). The *o*-chloranil group likely can change positions, alternating between each of the quinoline ligands, which could be one route to chemical environment exchange. The ^1^H NMR spectrum of **3** at −110 °C (utilizing a 60:27:13% by volume mixture of CD_2_Cl_2_/CCl_4_/CDCl_3_ as the solvent) shows the expected 2:1 ratio (Figure 5b) for the resonances due to quinoline groups; however, slow exchange was not achieved at −110 °C. Using the coalescence temperature (*T*_c_) of −70 °C (203 K) and the peak separation (∂ν) of 208.9 Hz of the most downfield proton resonances, we calculate an upper limit for the activation energy of the fluxional process for **3** as Δ*G* ^‡^ ≤ 7.5 kcal/mol using the method published by Shanan-Atidi and Bar-Eli for the treatment of differently populated doublets in variable temperature NMR (see Supporting Information Section 2 for more details).^28–30^ This low-barrier fluxional process for **3** might bear some resemblance to Berry pseudorotation in which group V compounds such as PF_5_ rapidly exchange axial and equatorial sites on the NMR time scale.^31^ A Berry-type pseudorotation would result in the exchange of quinoline ligands coordinated to Cu, as well as reorganization at Sb, resulting in coalesced NMR spectroscopy.

### Cyclic Voltammetry of {Q_3_Sb­(*o*-chlor)}­Cu­(OTf) (**3**) and (TMQA)­Cu­(OTf) (**5**)

The Cu^II^/Cu^I^ potentials for **3** and **5** were measured by using cyclic voltammetry in dichloromethane (DCM) (Figure 6). The *E*_1/2_ of the Cu^II^/Cu^I^ redox for **3** is 0.74 V vs Fc/Fc^+^ (Fc = ferrocene), while complex **5** displayed a significantly more negative Cu^II^/Cu^I^ redox potential at 0.07 V. The Cu^II/I^ redox couple for complex **5** is quasi-reversible with a large peak-to-peak separation (Δ*E*_p,a_ = 530 mV for **5** vs 140 mV for **3**). The solvent-dependent dimerization of related Cu­(I) tris­(2-pyridylmethyl)­amine complexes has been observed during cyclic voltammetry studies, especially in solvents less polar than acetonitrile (unfortunately, complex **3** decomposes in MeCN to form free ligand, see Figures S18 and S19). We were able to perform cyclic voltammetry of **3** and **5** in propylene carbonate; however, the oxidation of complex **3** is not reversible (Figures S26 and S27).^32^ Because of the quasi-reversibility of **5**, we also include the peak-to-peak separation between the oxidation maximum of both **3** and **5** (Δ*E*_p,a_ = 470 mV). This shift of *E*_1/2_ 670 mV (Δ*E*_p,a_ = 470 mV) is more positive for complex **3**, indicating less electron density on Cu compared to complex **5**. Also, we performed B3LYP-D3­(BJ)/def2-SVP DFT calculations to predict the redox potentials of both **3** and **5**, and the results agree with the relative experimental values. The calculations predict a Cu^II/I^ redox potential at 0.6 V for **3** and −0.3 V for **5** (versus Fc/Fc^+^).

**Figure 6:**
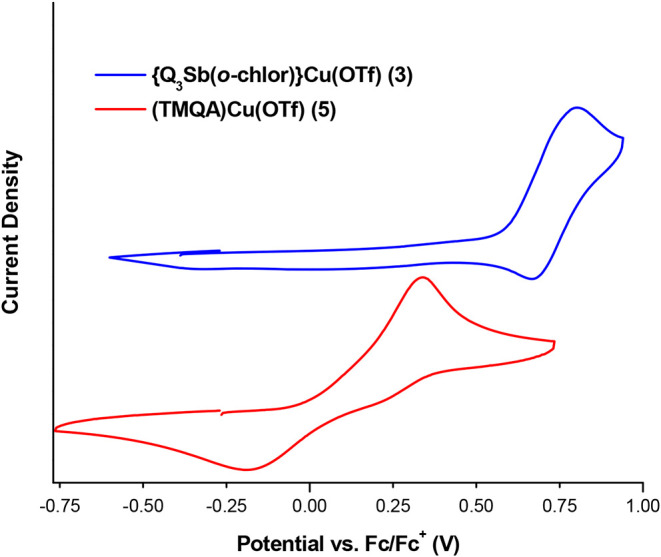
Cyclic voltammograms of {SbQ_3_(*o*-chlor)}­Cu­(OTf) (**3**) and (TMQA)­Cu­(OTf) (**5**) demonstrating the difference in Cu^II/I^ *E*_1/2_ potentials. Cyclic voltammetry was performed in a solution of 1 mM Cu complex **3** or **5** and 0.1 M TBAPF_6_ (tetrabutylammonium hexafluorophosphate) dissolved in 5 mL of DCM using a glassy carbon working electrode, Pt wire counter electrode, and a Ag/AgCl pseudoreference electrode (no *IR* correction). Ferrocene was added at the end of the experiment and used as an internal reference.

### XPS Experimental

X-ray photoelectron spectroscopy (XPS) was used to characterize the molecular complexes **1**, **2**, **3**, and **5**. Measurements were taken with a Scienta Omicron Multiprobe MXPS with a monochromatic Al Kα X-ray source (*h*ν = 1486.7 eV) referenced to the Au 4f_7/2_ core level at 84.0 eV. Survey spectra were measured with a pass energy of 80 eV, the individual core levels were recorded with a pass energy of 20 eV, and the valence band was measured with a 50 eV pass energy. The fine-grained powders were mounted on a strip of carbon tape, resulting in a coverage of >95% assessed from the intensity of the C–O-related peak at ∼288.5 eV. The molecules are insulating and require the use of a charge neutralizer during measurement. The neutralizer was set to an electron energy of 2 eV and an emission current of 4 μA, and no charge-induced shifts or peak distortions were seen with these conditions. Molecules were tested for radiation damage, and the workflow is included in the Supporting Information Section 5 and Figure S28. Essentially, survey spectra and individual peaks were compared at specific intervals throughout the XPS measurements to capture any changes in composition and chemical states. None of the molecules studied exhibited radiation damage.

The following core levels were recorded as needed for the respective molecule: Sb 3d_3/2_, Cu 2p_3/2_, Cl 2p, F 1s, O 1s, N 1s, C 1s, S 2p, and the valence band region. The Sb 3d_5/2_ and O 1s core levels overlap and therefore cannot be used for analysis. The survey spectra for each molecule are included in Supporting Information Section 5, Figure S30. The core levels were fit with Doniach–Šunjić (DS) line shape for Cu and Sb and Voigt functions for all other core levels in combination with a Shirley background. None of the molecules is metallic, and the DS function was only used to account for a small asymmetry in the Cu and Sb core levels, which is most likely due to defects or configurational variations. The quality of the fit was confirmed by assessment of the residuals. The molecule compositions with respect to Cu, Sb, Cl, F, N, and S are close to the given values, while O contributions are increased due to adsorbates, and compositions are summarized in the Supporting Information Section 5, Table S2.

The position of the core-level peaks is given with respect to the Fermi energy at a 0 eV binding energy as well as the valence band maximum (VBM). The use of the VBM as a reference is common in semiconductor studies and is independent of shifts of the Fermi level, which can be caused by defects in the band gap. The core-level positions for all elements are summarized in Table S3 in the Supporting Information. The position of the VBM was determined by applying the linear extrapolation method, in which the onset of the valence band density of states is extrapolated to the baseline intensity, which is then used as the VBM position.^43^ The use of the VBM as a reference leads to a rigid binding energy shift of −0.3, −1.3, −1.3, and −0.8 eV for all core levels in molecules 1, 2, 3, and 5, respectively, with an error of ±0.1–0.2 eV. This is illustrated in the Supporting Information Figure S31.

XPS and X-ray absorption near edge structure^44–46^ (XANES) have been used in the literature to study the charge state of the cation in molecular inorganic complexes. XPS is more surface sensitive compared to XANES, which is mitigated in our studies by the use of a glovebox and rapid transport in a dinitrogen atmosphere to the XPS instrument. Also, this rapid turnaround limits the degradation of sensitive compounds. XPS delivers quantitative information on the molecular composition, and every element can be studied, which includes the chemical shifts induced by manipulation of ligands. The chemical shift of the cation induced by a change in the charge state is discussed in comparison between compounds and the DFT calculations.

### X-Ray Photoelectron Spectroscopy of Q_3_Sb (**1**), Q_3_Sb­(*o*-chlor) (**2**), {SbQ_3_(*o*-chlor)}­Cu­(OTf) (**3**), and (TMQA)­Cu­(OTf) (**5**)

X-ray photoelectron spectroscopy (XPS) provides data relevant to transition metal charge, including examples of the use of XPS to study Cu^33^ and Ir molecular complexes.^34,35^ Given our interest in understanding the impact of the Cu–Sb­(V) bond in **3**, complexes **1**, **2**, **3**, and **5** were studied by XPS. The Sb 3d and O 1s core-level spectra and their corresponding fits are shown in Figure 7; Cu 2p and N 1s core-level spectra are displayed in Figure 8. Table 1 includes the binding energies and chemical shifts for complexes **1**–**3** and **5** from experiments and DFT calculated values obtained from orbital energies, adjusted to the highest occupied molecular orbital (HOMO) orbital energy level. All other core-level spectra, fit results, and binding energies for complexes **1**–**3** and **5** are included in the Supporting Information Section 5. Note that all binding energies cited in the main body of the manuscript are given with respect to the valence band maximum (VBM). Values with respect to the Fermi energy and the VBM are included in Supporting Information Section 5 for all elements.

**Figure 7:**
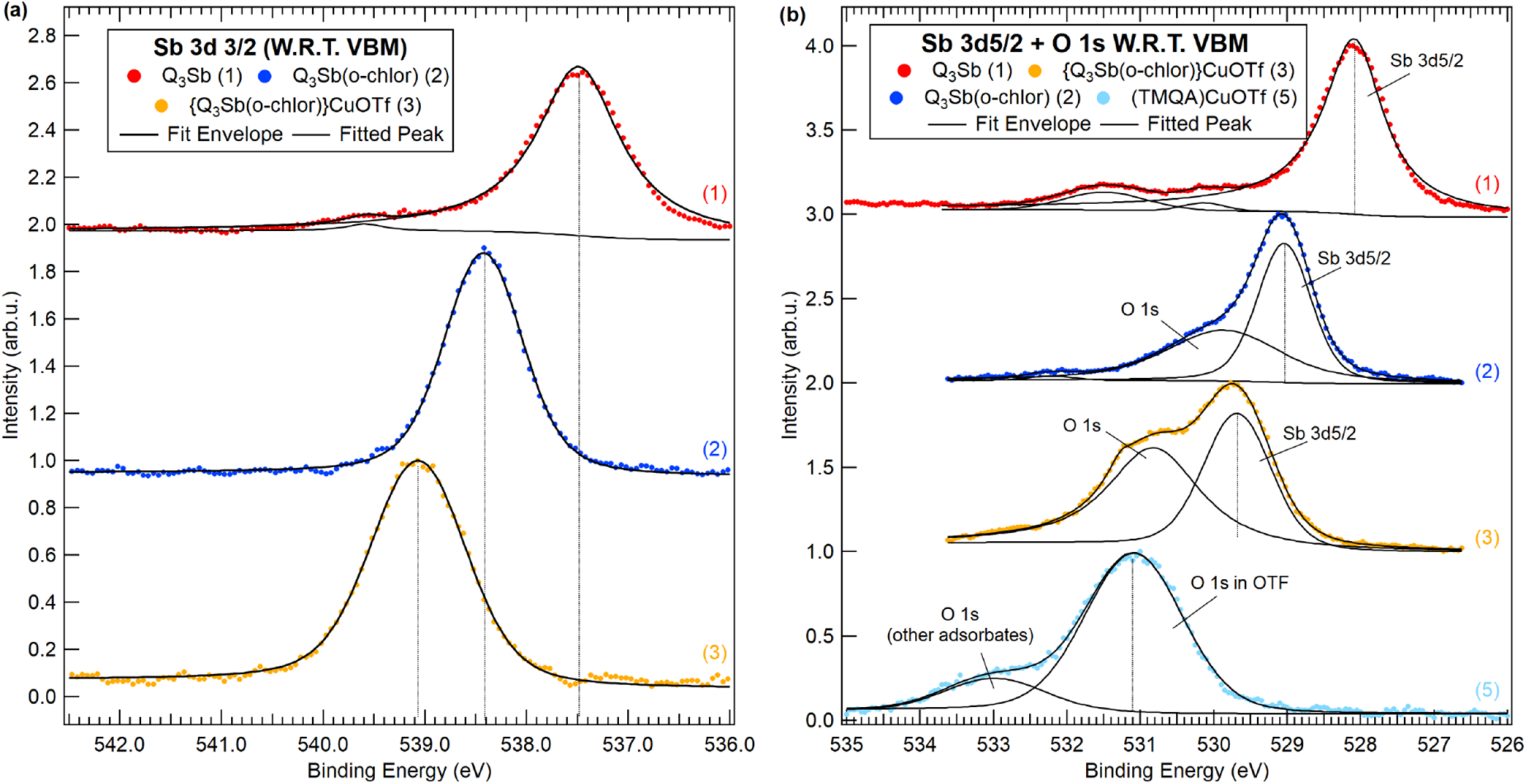
XPS spectra and fits for (a) the Sb 3d_3/2_ peak for **1**–**3** and (b) the Sb 3d_5/2_ peak and O 1s peak for **1**–**3** and **5** as applicable. The label W.R.T. VBM refers to the use of the valence band maximum as the reference energy.

**Figure 8:**
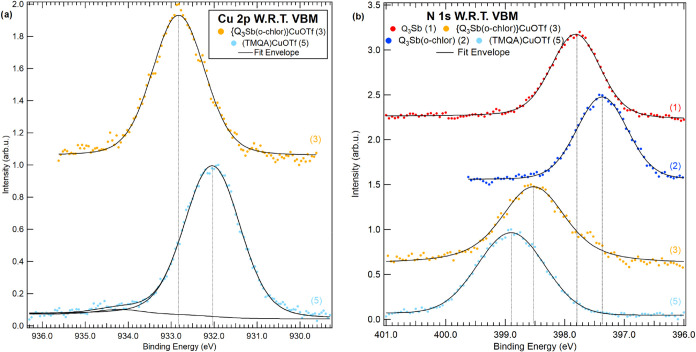
XPS spectra of (a) the Cu 2p peaks for **3** and **5** and (b) the N 1s peaks for **1**–**3** and **5**. The label W.R.T. VBM refers to the use of the valence band maximum as the reference energy.

**Table 1:** Calculated (B3LYP-D3­(BJ)) and Experimental Relative Binding Energies (eV) of Sb 3d_3/2_ and Cu 2p Orbitals of Q_3_Sb­(*o*-chlor) (**2**), {Q_3_Sb­(*o*-chlor)}­Cu­(OTf) (**3**), and (TMQA)­Cu­(OTf) (**5**)

relative binding energy (eV)^a^			
atom	complex	calculated	experimental (XPS)
Sb 3d_3/2_	Q_3_Sb(*o*-chlor) (**2**)	2.9^b^	0.9
	{Q_3_Sb(*o*-chlor)}Cu(OTf) (**3**)	3.0^b^	1.6
Cu 2p	(TMQA)Cu(OTf) (**5**)	–1.3^c^ (−2.0)^b^	–0.8

a Binding energies are reported relative to **1** for Sb 3d_3/2_ and to **3** for Cu 2p.

b Calculated with the x2c-SVPall basis set.

c Calculated with the def2-SVP basis set.

Figure 7a shows the Sb 3d_3/2_ core level, which is positioned at 537.5 eV for **1** and shifted significantly to higher binding energies by 0.9 eV for **2** (538.4 eV) and 1.6 eV for **3** (539.1 eV). The Sb 3d_3/2_ core level is used due to an overlap of the Sb 3d_5/2_ with the O 1s. These data are consistent with an increased cationic character from Sb^III^ for **1** to Sb^V^ for **2** and **3**. The 0.7 eV higher Sb 3d_3/2_ binding energy for **3** compared to **2** is likely related to Cu donating less electron density to Sb^V^ for **3** compared to N-based electron donation through pnictogen bonding as observed in the crystal structure of **2**. Also, Cu coordination might result in a net electron-withdrawing effect from the quinoline ligands, leading to a higher Sb binding energy. Consistent with the experimental measurements, B3LYP-D3­(BJ)/def2-SVP energies, adjusted to the HOMO level for each complex, showed a 0.3 eV higher binding energy for **3** compared to **2**. While the def2-SVP basis provided reasonable values for Cu energies, this basis set gave significant error for the Sb 3d_3/2_ binding energies. A survey of basis sets showed that x2c-SVPall provided the most reasonable values but still with overestimated binding energies compared to experiment and with only a small difference between complexes **2** and **3**. For all basis sets examined, **3** has a larger binding energy than **2**. The difference between experimental and calculated binding energies can also be partially attributed to different choices of origin in the energy scale and the challenges in fully replicating the final state effects and contributions of core hole relaxation within the DFT calculation. By the same token, XPS core-level binding energies are not necessarily linearly dependent on the oxidation state of the cation and can, especially for the large organic molecules, be influenced by remote dipoles^36,37^ modulating the binding energies and hence the measured chemical shift.

For **1**, a small peak is visible at ∼539.5 eV and is assigned to an impurity and compatible with a small amount of oxidized O = SbQ_3_ upon exposure to air. The 3d_3/2_ shifts are mirrored in the Sb 3d_5/2_ core level, which overlaps with that of the O 1s (Figure 7b). Both Sb 3d_5/2_ and O 1s move to higher binding energy for complex **3** compared to **1** and **2**, similar to observed shifts for the Sb 3d_3/2_ core level. Sb 3d_5/2_ is assigned from the position of the Sb 3d_3/2_ peak using the spin-orbit splitting of 9.4 eV for complexes **1** to **3**. The lower intensity of the O 1s compared to the Sb 3d_5/2_ peak is due to the 5 times larger cross section of the Sb 3d_5/2_ core level.^38,39^ A table with molecule compositions is included in Supporting Information Section 5. Oxygen in complex **5** is at 531.1 eV, and the peak at 533.0 eV for **5** corresponds to the oxygen adsorbates. The peak at 529.9 eV for **2** is assigned to the oxygen atoms of the coordinated *o*-chloranil ligand. The two contributions of oxygen atoms from the triflate and *o*-chloranil cannot be separated for **3**, for which both are overlapped and are chemically shifted toward each other, leading to a relatively broad peak. All other contributions are minor and attributed to adsorbates or contamination.

Figure 8 includes the Cu 2p and N 1s core levels. Only molecules **3** and **5** include Cu atoms, and in both cases Cu is bonded to the electron-deficient triflate group. Comparing **3** to **5**, the Cu 2p_3/2_ core level is shifted from 932.8 eV by 0.8 eV to lower binding energy, indicating a more electron-rich Cu center in complex **5** (932.0 eV) compared to complex **3**. These data are consistent with an electron-withdrawing effect of Sb­(V) in **3** compared to a donating N bonded to Cu in **5**.

DFT calculations predict a 1.3 eV lower relative binding energy of the Cu 2p core level for complex **5** vs **3** in close agreement with experimentally determined binding energies. Figure 8 includes the core levels for N 1s for **1**–**3** and **5**. All nitrogen atoms in **1** have the same bonding environment as part of a quinoline ring system and are positioned at 397.8 eV. The chemical shift of N 1s toward higher binding energy is indicative of diminished electron density at the N-site, which agrees with this interpretation. Surprisingly, the nitrogen binding energy decreases by 0.4 eV from **1** to **2**, given the electron-withdrawing effect of Sb­(V); however, the geometry of **1** is different from **2**, which could lead to a more complicated interpretation than solely inductive withdrawing effects.

### DFT Bonding Analysis of {Q_3_Sb­(*o*-chlor)}­Cu­(OTf) (**3**) and (TMQA)­Cu­(OTf) (**5**)

We generated and inspected the molecular orbitals as well as the natural localized molecular orbitals (NLMOs) of **3** and **5** to evaluate and compare possible Cu to Sb orbital bonding (Figure 9). The molecular orbital corresponding to the Cu–Sb interaction of complex **3** shows only a little bonding between Cu and Sb. In contrast, the NLMO reveals Cu–Sb interaction, but it is heavily skewed toward Cu, with 87% assigned to Cu and 8% assigned to Sb. A similarly skewed bonding was found with the intrinsic bond orbitals. Because the Cu–Sb bond in **3** is skewed toward Cu, the oxidation states can be assigned as Cu­(I) and Sb­(V) with a weak Cu → Sb Z-type interaction. The close distance between Cu and Sb in **3** (2.659 Å and 2.6761(11) for the calculated and single-crystal X-ray structure, respectively), is therefore likely due to the constraining bridging quinolines as opposed to a significant Z-type interaction. Consistent with the single-crystal X-ray structure for complex **5**, the DFT optimized structure shows the triflate counterion disconnected from the Cu center (the Cu–O distances are 3.676 vs 4.428(5) Å for calculated and single-crystal X-ray structure {shortest Cu–O bond in the asymmetric unit}, respectively). A close match was found for the Cu–OTf distance of **3** between calculated (2.121 Å) and single-crystal X-ray structures (2.198(4) Å). As expected, analysis of the Cu–N bridgehead bond of **5** shows the expected L-type ligand donation and a dative covalent bond with Cu–N bond distances of 2.282 vs 2.209(5) Å for calculated and single-crystal X-ray structures, respectively.

**Figure 9:**
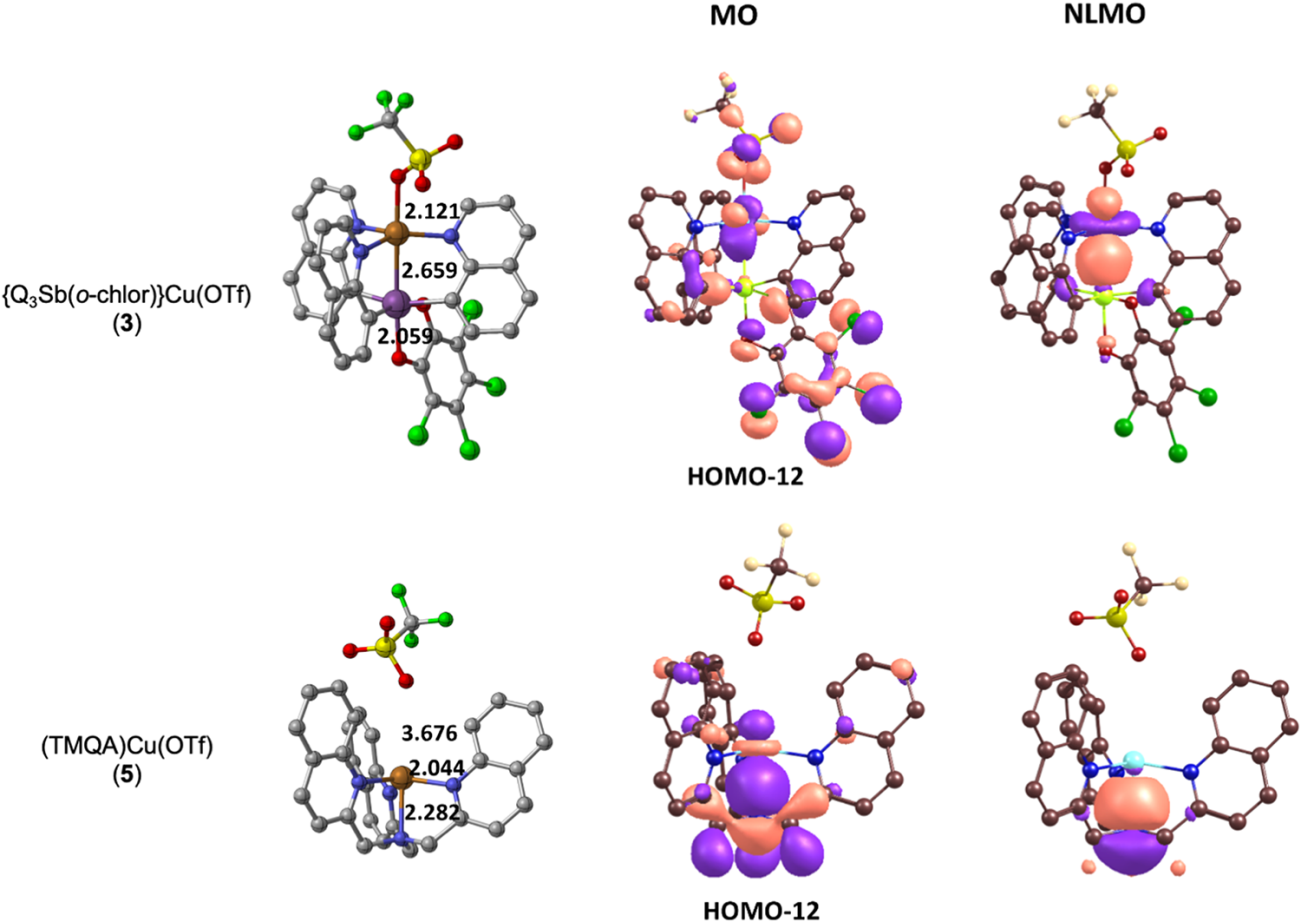
Comparison of orbital bonding for complexes {Q_3_Sb­(*o*-chlor)}­Cu­(OTf) (**3**) and (TMQA)­Cu­(OTf) (**5**). MO = fully delocalized Kohn–Sham DFT orbitals. NLMO is natural localized mole*c*ular orbitals generated with the natural bond orbital (NBO) program.

## Summary and Conclusions

We have isolated a Cu–Sb complex by reacting a soluble Cu­(I) triflate salt with a Lewis acidic Sb­(V) precursor. We characterized the complex via ^1^H, ^13^C­{^1^H} and ^19^F­{^1^H} NMR spectroscopy, elemental analysis, and single-crystal X-ray diffraction. Variable temperature ^1^H NMR studies of both the Sb­(V) compound **2** and the Cu–Sb complex **3** indicate fluxionality at room temperature. The impact of the Sb­(V) ligand **2** coordinated to Cu­(I) was probed via cyclic voltammetry and XPS and compared to a non-Sb-containing complex **5**. Cyclic voltammetry studies of **3** and **5** show less electron density on Cu for **3** with a 670 mV shift to higher potentials for the Cu^II/I^ *E*_1/2_ for **3** compared to **5** (Δ*E*_p,a_ = 470 mV). XPS studies further demonstrate a more electron-poor Cu center in **3** than in **5**, with a shift to a higher binding energy of the Cu 2p core level by 0.8 eV. Both cyclic voltammetry and XPS studies were coupled with DFT studies. Inspection of the molecular orbitals and localized orbitals, natural bonding orbitals (NBOs), suggests a weak Cu → Sb Z-type interaction.

## Experimental Section

### General Information

All reactions were performed in a N_2_-filled glovebox. The glovebox was purged periodically, ensuring <20 ppm of either O_2_ or H_2_O. Tri­(quinolin-8-yl)­stibane (Q_3_Sb) was synthesized on a Schlenk line under N_2_ as previously reported.^10^ All NMR reactions were performed using medium-wall precision low-pressure/vacuum (LPV) NMR tubes. Tetrahydrofuran (THF) and diethyl ether (Et_2_O) were dried via a potassium-benzophenone/ketyl still under a N_2_ atmosphere or by using a solvent purification system with activated alumina and stored over activated 4 Å molecular sieves inside a glovebox. Pentane and methylene chloride were dried using a solvent purification system with activated alumina and stored under activated 4 Å molecular sieves inside a N_2_-filled glovebox. Chloroform-*d* and methylene chloride-*d*_2_ were stored over activated 4 Å molecular sieves inside a glovebox. The TMQA ligand **4** was synthesized as previously described.^26^ All other chemicals were purchased from commercial sources and used as received.

NMR spectra were recorded on a Varian VNMRS 600 MHz or a Bruker Avance III 800, 600, or 400 MHz spectrometer. All reported chemical shifts are referenced to residual ^1^H resonances (^1^H NMR) or ^13^C resonances (^13^C­{^1^H} NMR). ^1^H NMR: chloroform-*d* 7.26 ppm; methylene chloride*-d*_2_ 5.32 ppm; *d*_3_-MeCN 1.94 ppm. ^13^C­{^1^H} NMR: chloroform-*d* 77.2 ppm; methylene chloride*-d*_2_ 53.8 ppm.^40^ ^19^F­{^1^H} NMR spectra were referenced to hexafluorobenzene δ −164.9 ppm using an external standard. Elemental analyses were performed by the University of Virginia Chemistry Department Elemental Analysis Facility using a PerkinElmer CHNS-O series II analyzer. Cyclic voltammetry was performed using a Pine WaveNow potentiostat inside a N_2_-filled glovebox. Cyclic voltammetry was performed in a solution of 1 mM Cu complex (**3** or **5**) and 0.1 M TBAPF_6_ dissolved in 5 mL of DCM using a glassy carbon working electrode, a Pt wire counter electrode, and an Ag/AgCl pseudoreference electrode (no *IR* correction). Ferrocene was added at the end of the experiment and used as an internal reference.

### Computational Details

Geometry optimizations were completed with B3LYP-D3­(BJ)/def2-SVP in Gaussian 16.^41^ During geometry optimization, solvent effects were incorporated using the conductor-like polarizable continuum model (CPCM) method for dichloromethane (DCM). All of the stationary points were characterized as a minimum using vibrational frequency analysis. For the CV calculations of Cu, ferrocene was used as a reference electrode. NLMO calculations were performed using the NBO program,^42^ as implemented in Gaussian 16. For XPS, the valence band maximum (VBM) energies were adjusted to be relative to the highest occupied molecular orbital (HOMO) energy.

### Synthesis and Characterization

#### Q_3_Sb­(*o*-chlor) (**2**)

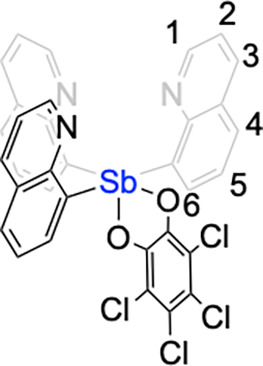



To a round-bottom flask containing 50 mL of DCM, 200 mg (0.39 mmol) of Q_3_Sb were added. To the same flask, 97 mg (0.39 mmol) of *o*-chloranil was added to form a red/orange solution. Shortly after the addition of *o*-chloranil, a yellow precipitate formed. The reaction was stirred for 2 h. The solvent was then evaporated via vacuum until ∼10 mL remained; then, pentane was added to give an off-white precipitate. The white solid was then washed with further pentanes and dried to yield the product as an off-white solid (253 mg, 85%). ^1^H NMR (800 MHz, CD_2_Cl_2_) δ 8.79 (dd, ^3^ *J*_HH_ = 7, 1 Hz, 3H, 1-position), 8.65 (dd, ^3^ *J*_HH_ = 4, 2 Hz, 3H, 4- or 6-position), 8.16 (dd, ^3^ *J*_HH_ = 8, 2 Hz, 3H, 4- or 6-position), 7.85 (dd, ^3^ *J*_HH_ = 8, 1 Hz, 3H, 3-position), 7.64 (dd, ^3^ *J*_HH_ = 8, 7 Hz, 3H, 2-position), 7.34 (dd, ^3^ *J*_HH_ = 8, 4 Hz, 3H). ^1^H NMR (600 MHz, CD_3_CN) δ 8.77 (d, ^3^ *J*_HH_ = 7 Hz, 3H), 8.67 (dd, ^3^ *J*_HH_ = 4, 2 Hz, 3H), 8.28 (dd, ^3^ *J*_HH_ = 8, 2 Hz, 3H), 7.95 (d, ^3^ *J*_HH_ = 8 Hz, 3H), 7.68 (t, ^3^ *J*_HH_ = 8 Hz, 3H), 7.43 (dd, ^3^ *J*_HH_ = 8, 4 Hz, 3H). ^13^C­{^1^H} NMR (201 MHz, CD_2_Cl_2_) δ: 150.7, 149.1, 148.5, 147.3, 136.7, 135.1, 129.3, 128.7, 127.6, 121.8, 118.7, 115.8. Anal. Calcd for C_33_H_18_Cl_4_N_3_O_2_Sb: C, 52.70; H, 2.41; N, 5.59. Found: C, 52.78; H, 2.56; N, 5.26.

#### {Q_3_Sb­(*o*-chlor)}­Cu­(OTf) (**3**)

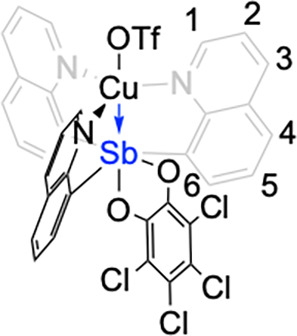



To a round-bottom flask inside a N_2_-filled glovebox, 105 mg (0.14 mmol) Q_3_Sb­(*o*-chlor) were suspended in ∼25 mL of THF. In a separate vial, 36 mg (0.07 mmol) of [CuOTf]_2_·C_6_H_6_ were suspended in ∼5 mL of THF and mixed via pipette agitation to produce a red suspension. The suspension of [CuOTf]_2_·C_6_H_6_ was then added dropwise to the vigorously stirring suspension of **2**, resulting in a yellow solution once all of the [CuOTf]_2_·C_6_H_6_ had been added. After stirring for 5.5 h, the solvent was evaporated under vacuum, leaving a yellow powder. The yellow powder was washed with diethyl ether and dried, leaving a yellow powder as product 98 mg (73% yield). Note: a small intractable impurity was observed in the aromatic region that could be washed out with THF at the expense of yield (45% isolated yield following the THF wash). ^1^H NMR (600 MHz, CD_2_Cl_2_) δ 9.65 (dd, ^3^ *J*_HH_ = 5, 2 Hz, 3H, 1-position), 8.46 (dd, ^3^ *J*_HH_ = 8, 2 Hz, 3H, 3-position), 8.25 (dd, ^3^ *J*_HH_ = 7, 1 Hz, 3H, 4- or 6-position), 7.97 (dd, ^3^ *J*_HH_ = 8, 1 Hz, 3H, 4- or 6-position), 7.70 (dd, ^3^ *J*_HH_ = 8, 5 Hz, 3H, 2-position), 7.63 (dd, ^3^ *J*_HH_ = 8, 7 Hz, 3H, 5-position). ^13^C­{^1^H} NMR (201 MHz, CD_2_Cl_2_) δ 154.7, 150.3, 146.4, 144.7, 140.3, 134.0, 131.1, 130.8, 128.4, 123.2, 120.3, 117.6. ^19^F NMR (565 MHz, CDCl_3_) δ −76.4. Anal. Calcd for C_34_H_18_Cl_4_CuF_3_N_3_O_5_SSb·0.06­(Et_2_O): C, 42.44; H, 1.93; N, 4.34. Found: C, 42.04; H, 2.11; N, 4.63. Note: 0.06 equiv of Et_2_O was present in the sample as quantified via ^1^H NMR (Supporting Information Figure S9).

#### (TMQA)­Cu­(OTf) (**5**)

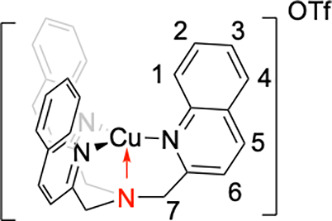



TMQA was suspended in ∼5 mL of DCM in a round-bottom flask inside a N_2_-filled glovebox. In a separate vial, 40 mg (0.07 mmol) of [CuOTf]_2_·C_6_H_6_ were suspended in ∼5 mL of DCM. The mixture of [CuOTf]_2_·C_6_H_6_ was added dropwise to the vigorously stirring suspension of TMQA, turning to a yellow solution once all [CuOTf]_2_·C_6_H_6_ was added. After stirring for 8 h, the solution was orange, and then the solvent was evaporated under vacuum to leave an orange powder. The orange powder was crystallized with DCM/diethyl ether slow evaporation in the freezer (−32 °C), leaving orange crystals as a product (50 mg, 56% yield). ^1^H NMR (800 MHz, CD_2_Cl_2_) δ 8.80 (d, ^3^ *J*_HH_ = 8 Hz, 3H, 1- or 4-position), 8.30 (d, ^3^ *J*_HH_ = 8 Hz, 3H, 5- or 6-position), 8.00 (t, ^3^ *J*_HH_ = 8 Hz, 3H, 2- or 3-position), 7.90 (d, ^3^ *J*_HH_ = 8 Hz, 3H, 1- or 4-position), 7.65 (t, ^3^ *J*_HH_ = 8 Hz, 3H, 2- or 3-position), 7.50 (d, ^3^ *J*_HH_ = 8 Hz, 3H, 5- or 6-position), 4.62 (s, 6H, 7-position). ^13^C­{^1^H} NMR (201 MHz, CD_2_Cl_2_) δ: 158.6, 145.8, 138.5, 131.7, 129.7, 129.2, 128.6, 128.0, 121.6, 61.4. Anal. Calcd for C_31_H_24_CuF_3_N_4_O_3_S: C, 57.01; H, 3.70; N, 8.58. Found: C, 57.25; H, 3.38; N, 8.36.

## Supplementary Material





## Notes

Experimental procedures and details, NMR spectra, and crystal structure data (PDF)
Cartesian coordinates for the calculated structures (XYZ)
