# Supplementary material for: Synthesis and Characterization of a Copper Complex Supported by a Z‑type SbV Ligand: XPS and DFT Study of Electronic Structure
Source: Organometallics. 2026 Feb 5;45(4):438–48. doi: 10.1021/acs.organomet.5c00420 (PMC12933881; doi:10.1021/acs.organomet.5c00420)
Supplement: Supplementary file 1 [file om5c00420_si_001.pdf]

Supporting Information
for

Synthesis and Characterization of a Copper Complex Supported by a Z-type Sb^V Ligand: XPS and DFT Study of Electronic Structure

Christopher K. Webber,[†] Macarena G. Alférez,[†] Farzad Bastani,[$] Jugal Kumawat,[§] Fanji Kong,[†] Zoë M. Gehman,[†] Xinrui Ou,[†] Diane A. Dickie,[†] Daniel H. Ess,[*§] Petra Reinke,[*$] T. Brent Gunnoe[*†]

*Corresponding authors. Email: tbg7h@virginia.edu, dhe@byu.edu, pr6e@virginia.edu

[†]Department of Chemistry, University of Virginia; Charlottesville, Virginia 22904, United States.

[$]Department of Materials Science and Engineering, University of Virginia; Charlottesville, Virginia 22904, United States.

[§]Department of Chemistry and Biochemistry, Brigham Young University; Provo, Utah 84604, United States.

Table of Contents

1. NMR Spectra 3
2. Calculation of Activation Energy Barrier for {Q_3Sb(o-chlor)}Cu(OTf) 21
3. Cyclic Voltammetry Studies 22
4. Crystallographic Data 27
5. Details of XPS Experiments 30
7. References 36

1. NMR Spectra

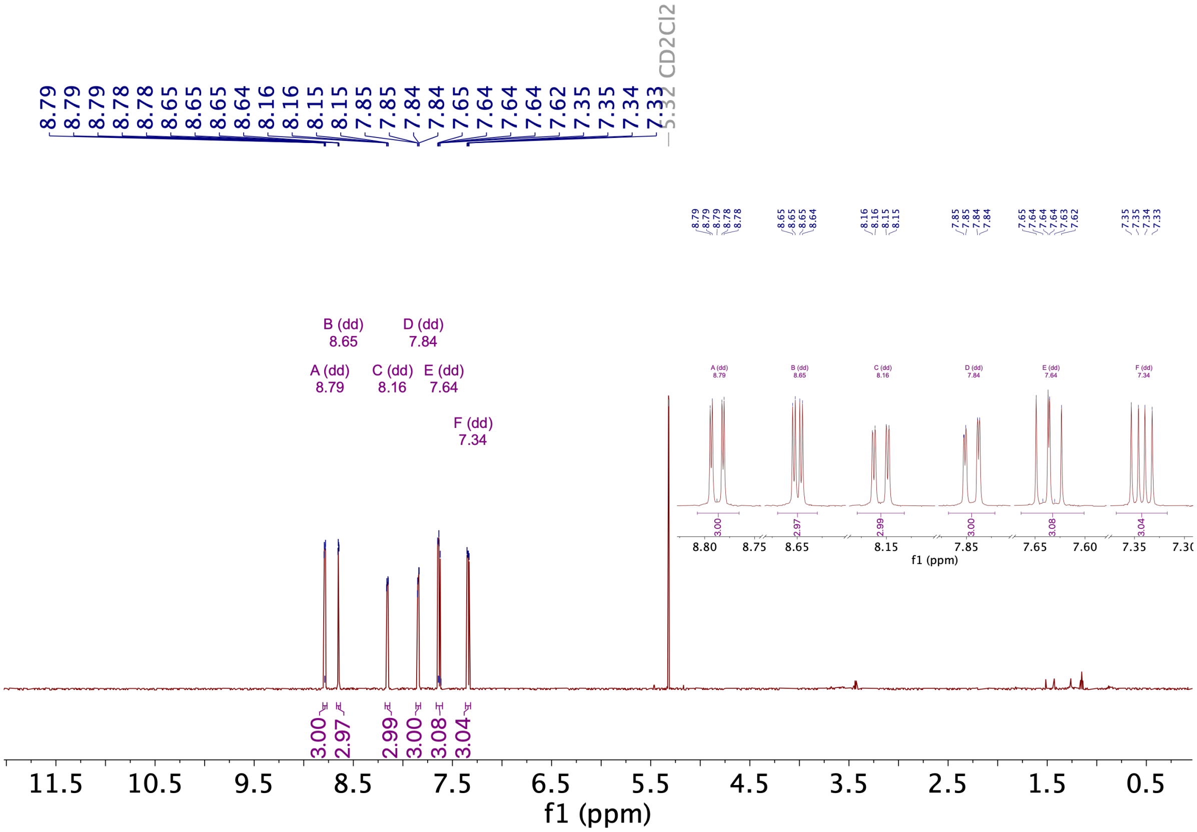

Figure S1. ^{1}H NMR spectrum of Q_3Sb(*o*-chlor) (**2**) (600 MHz, CD_2Cl_2). *Note*: trace solvent impurities are present in the upfield region.

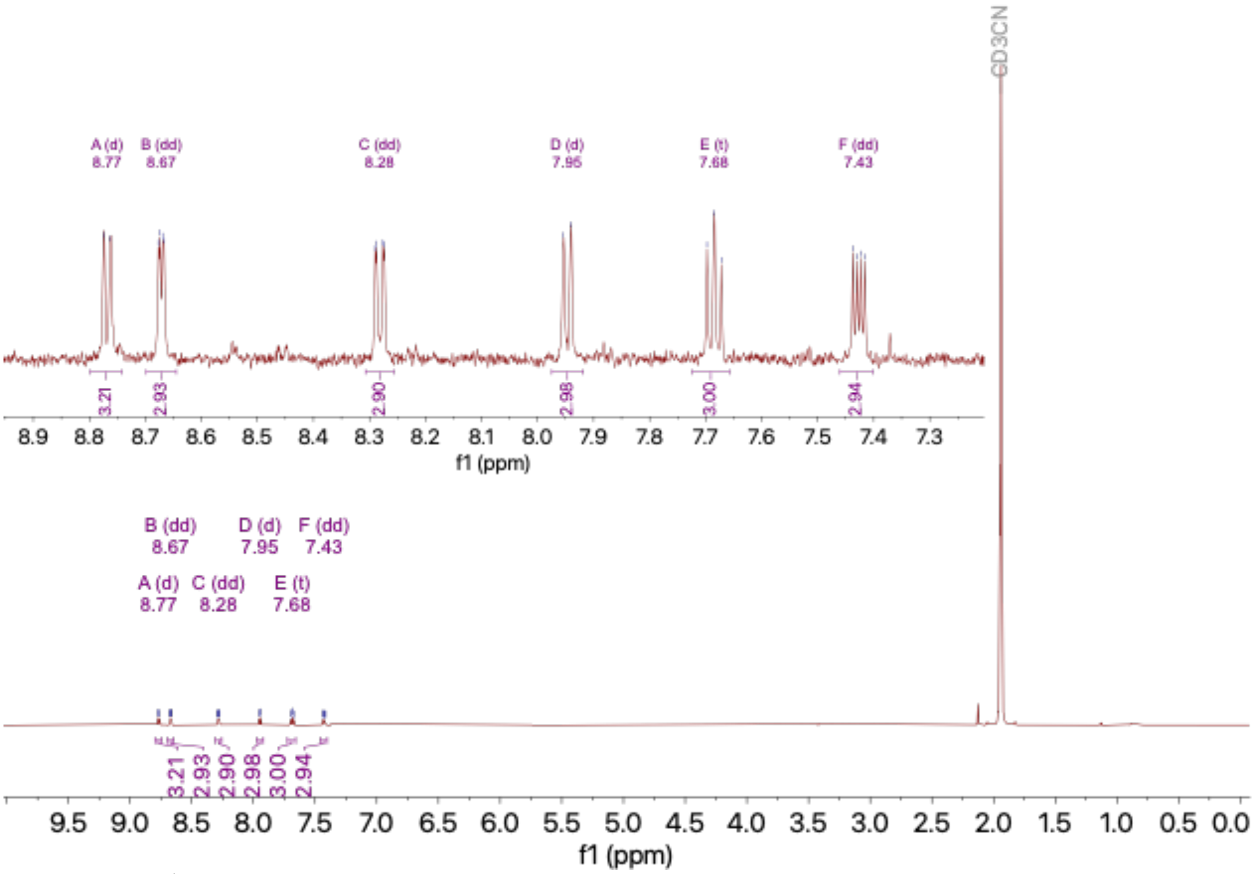

Figure S2. ^{1}H NMR spectrum of Q_3Sb(*o*-chlor) (**2**) (600 MHz, d_3-MeCN). *Note*: trace water impurity is present in the upfield region.

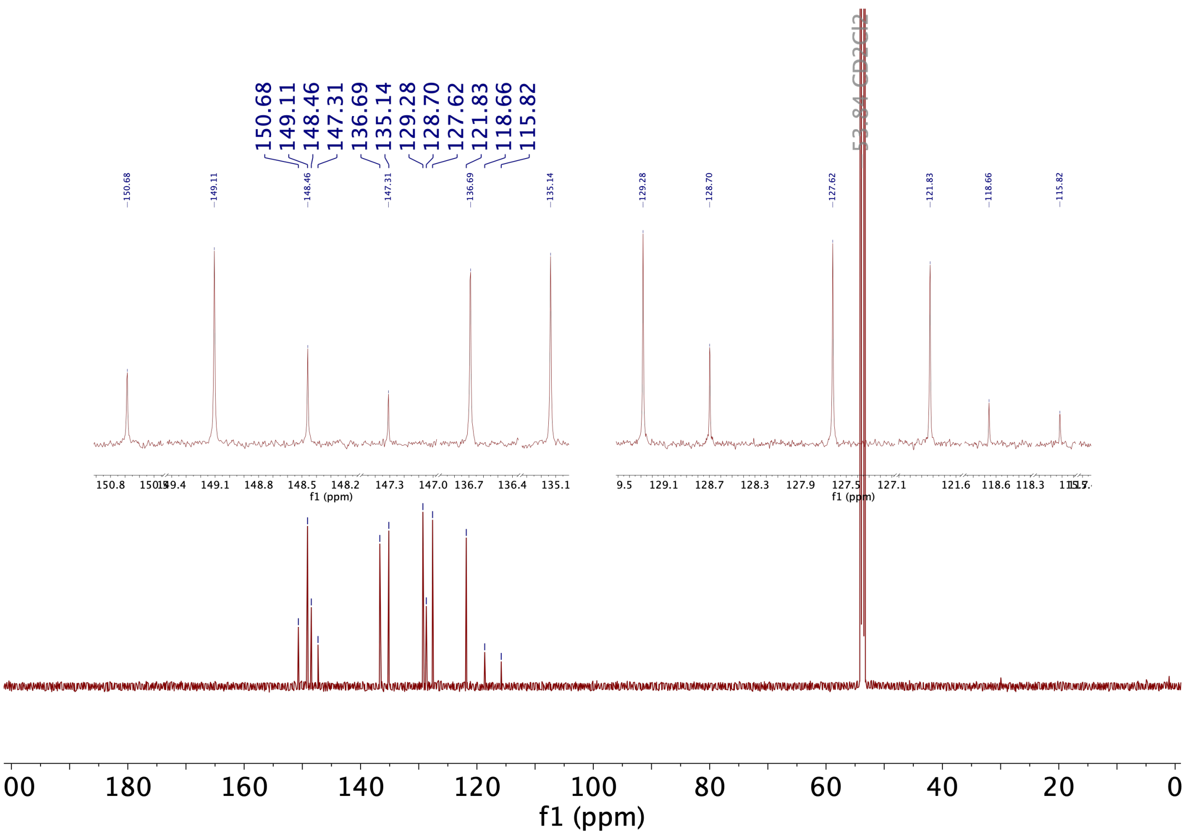

Figure S3. $^{13}C\{^{1}H\}$ NMR spectrum of Q_3Sb(o-chlor) (**2**) (201 MHz, CD_2Cl_2).

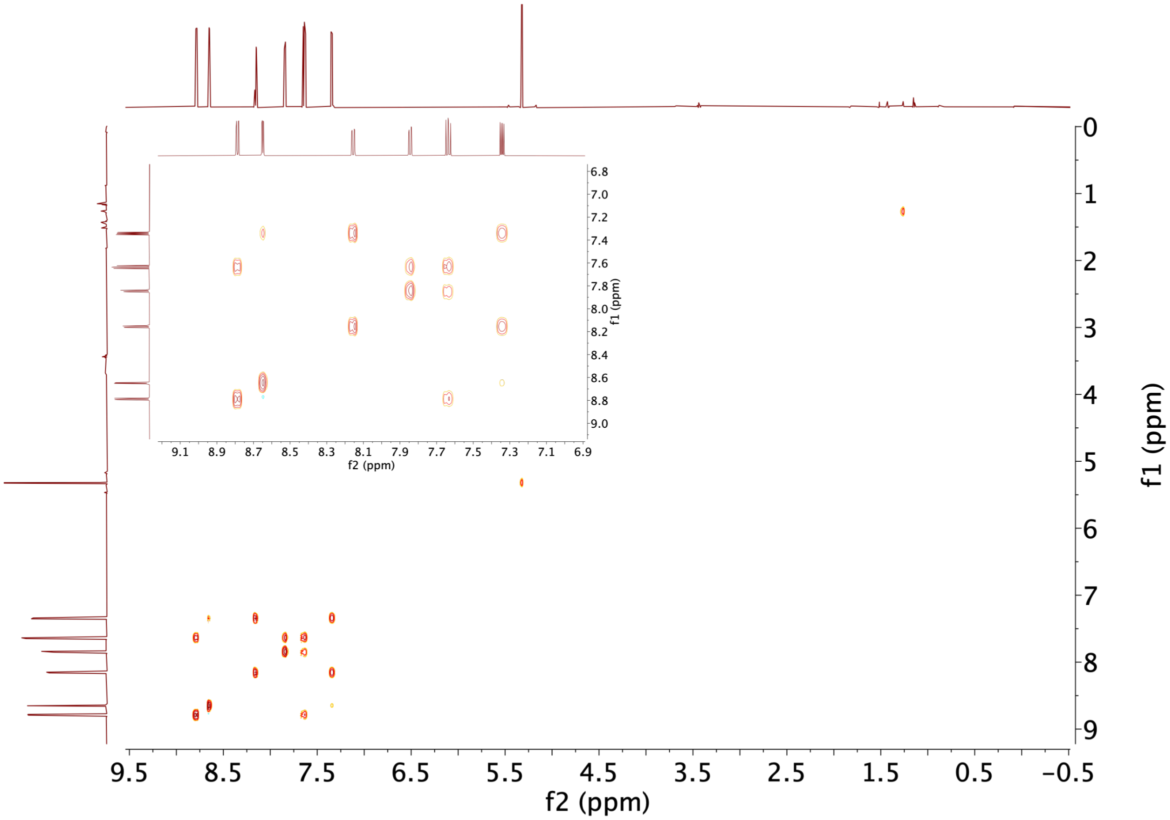

Figure S4. COSY NMR spectrum of Q_3Sb(o-chlor) (**2**) (400 MHz, CD_2Cl_2).

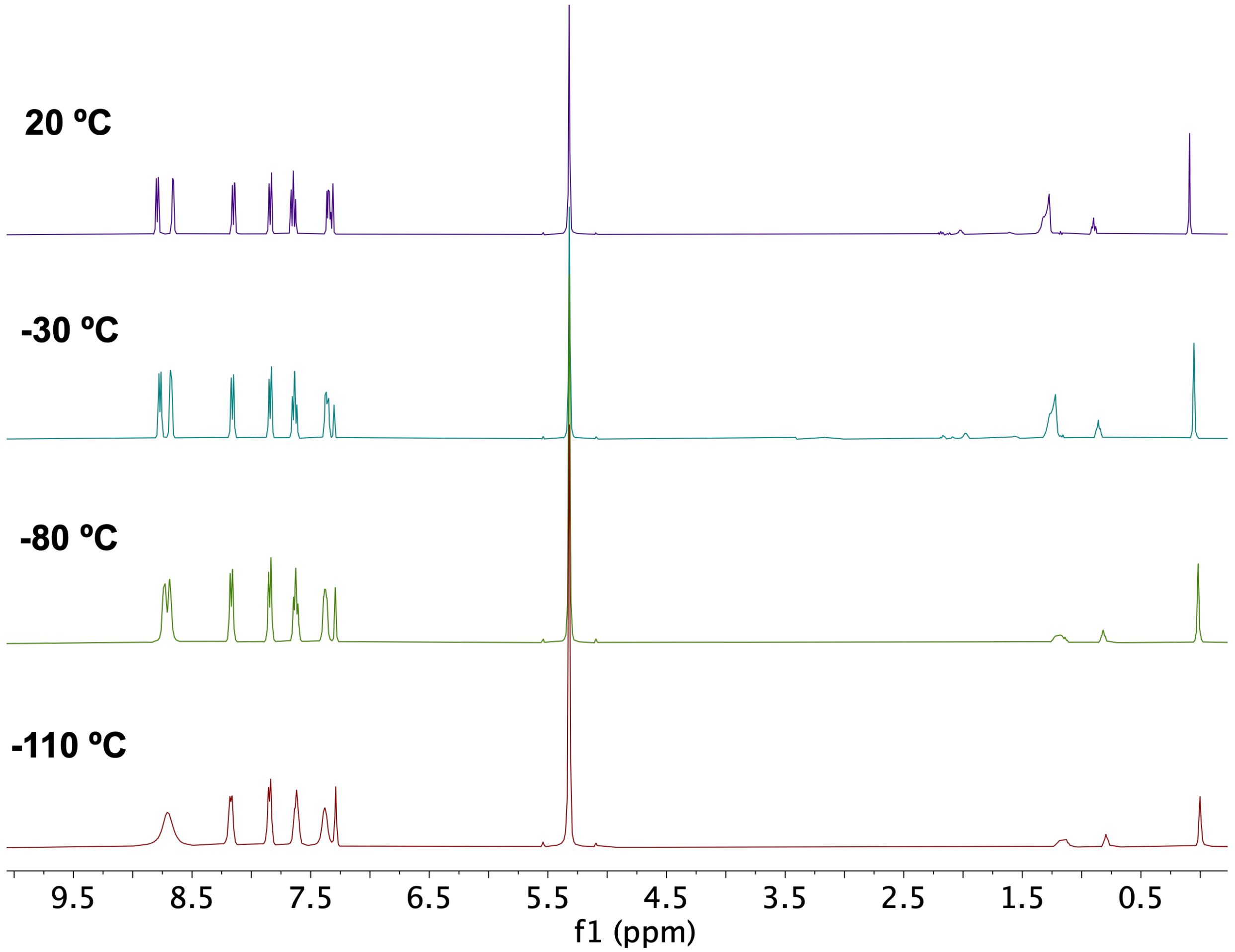

Figure S5. ^{1}H NMR spectra of Q_3Sb(*o*-chlor) (**2**) (60:27:13% by volume mixture of CD_2Cl_2:CCl_4:$CDCl_3$, 400 MHz) at various temperatures. *Note:* trace pentanes and silica grease impurities are present in the upfield region.

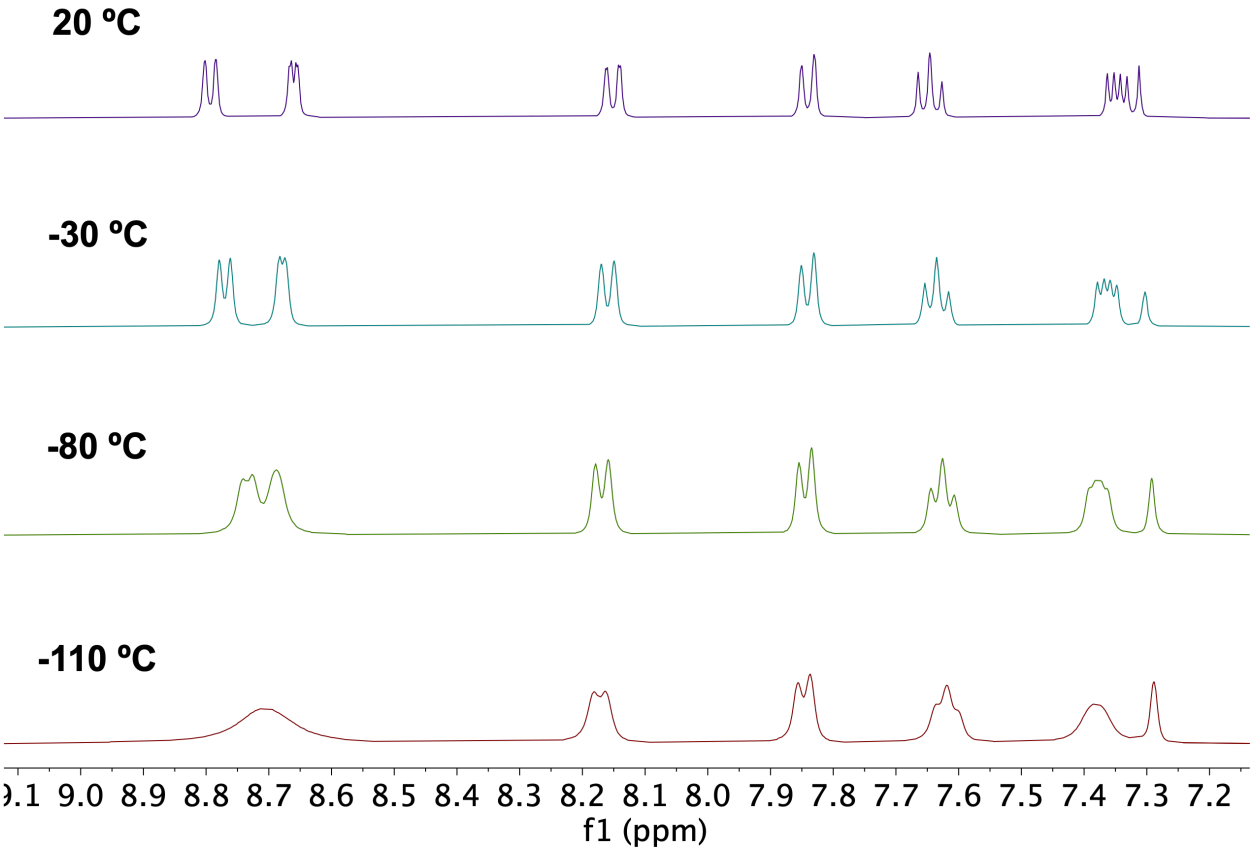

Figure S6. ^{1}H NMR spectra (aromatic region) of Q_3Sb(*o*-chlor) (**2**) (60:27:13% by volume mixture of $CD_2Cl_2:CCl_4:CDCl_3$, 400 MHz) at various temperatures. *Note:* trace pentanes and silica grease impurities are present in the upfield region.

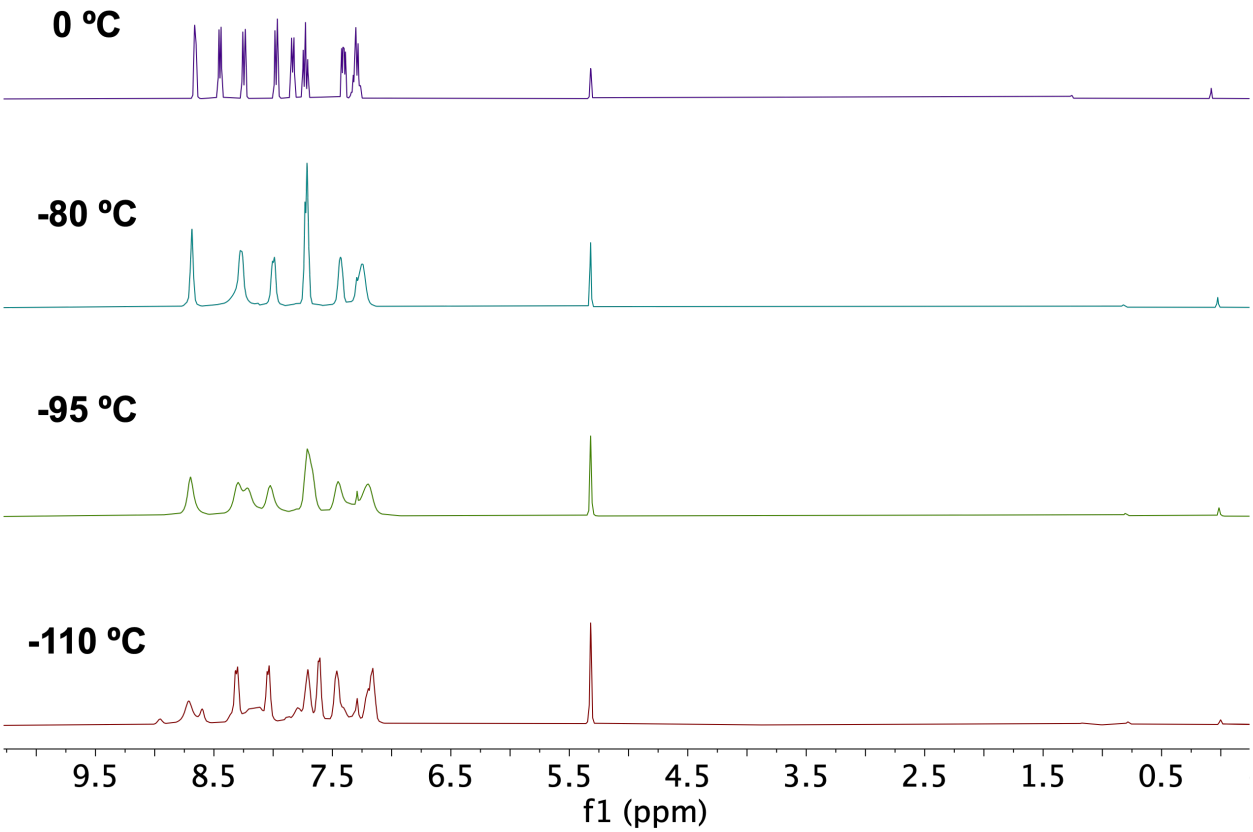

Figure S7. ^{1}H NMR spectra of Q_2SbPh(*o*-chlor) (60:27:13% by volume mixture of $CD_2Cl_2:CCl_4:CDCl_3$, 400 MHz) at various temperatures. *Note:* trace silica grease impurities are present in the upfield region.

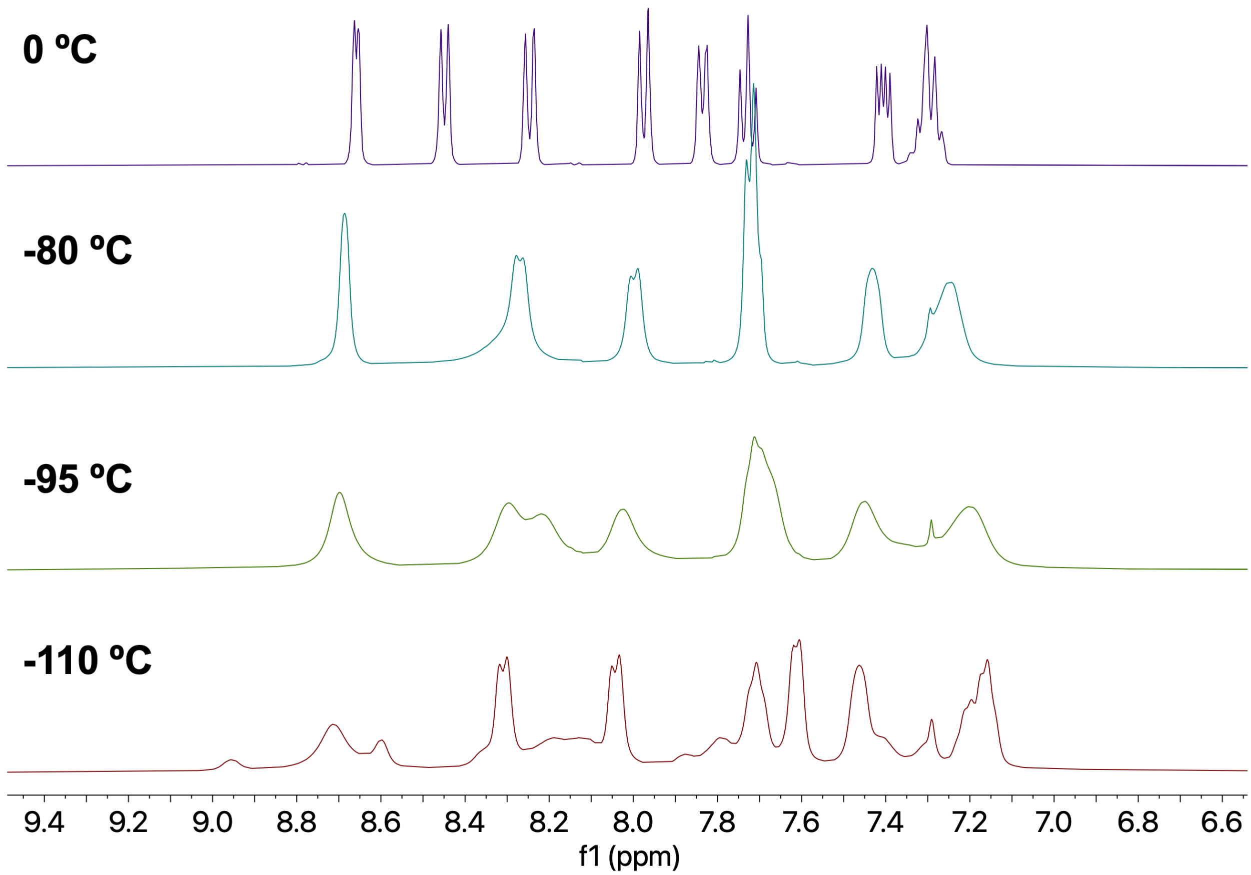

Figure S8. ^{1}H NMR spectra (aromatic region) of Q_2SbPh(*o*-chlor) (60:27:13% by volume mixture of CD_2Cl_2:CCl_4:$CDCl_3$, 400 MHz) at various temperatures.

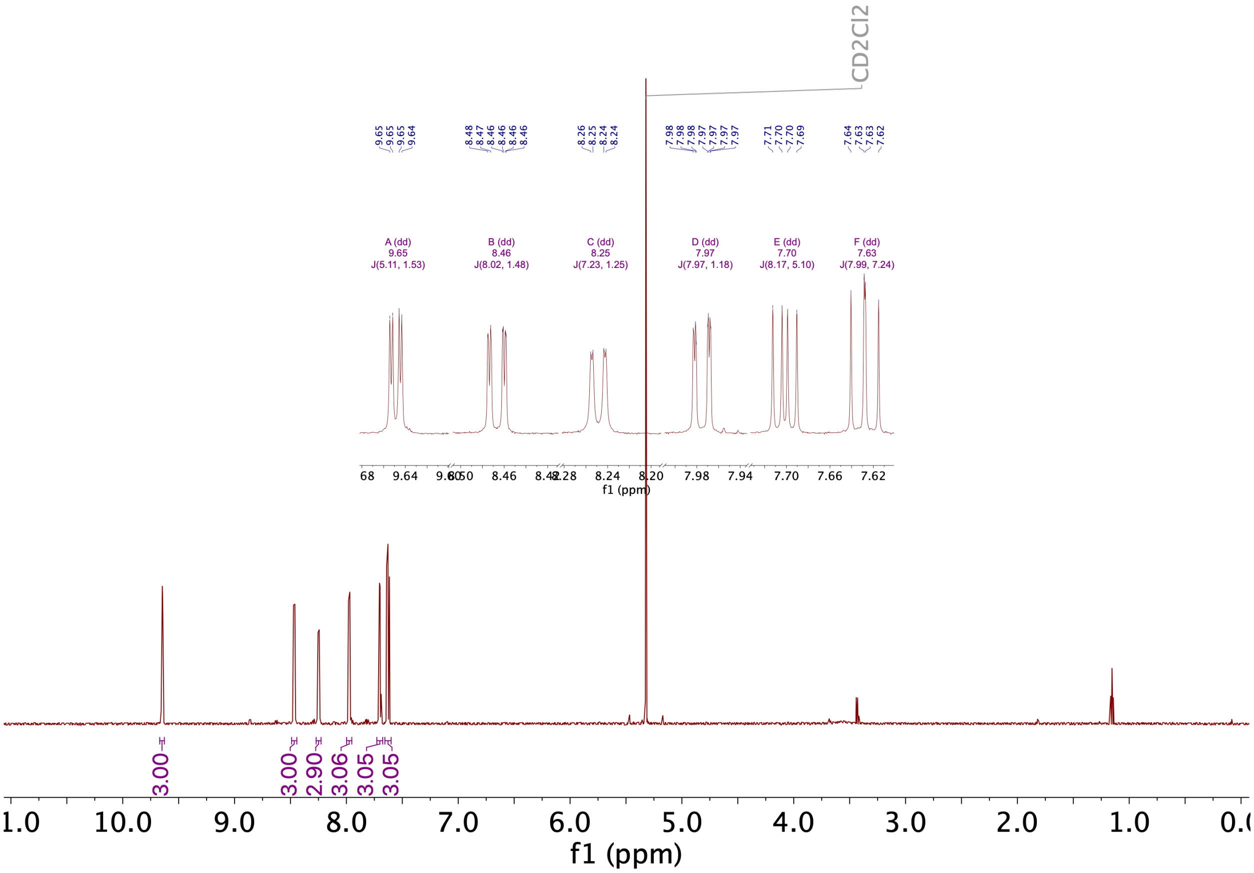

Figure S9. ^{1}H NMR spectrum of {Q_3Sb(*o*-chlor)}Cu(OTf) (**3**) (600 MHz, CD_2Cl_2). *Note*: approximately 0.06 equivalents of diethyl ether are present in spectrum.

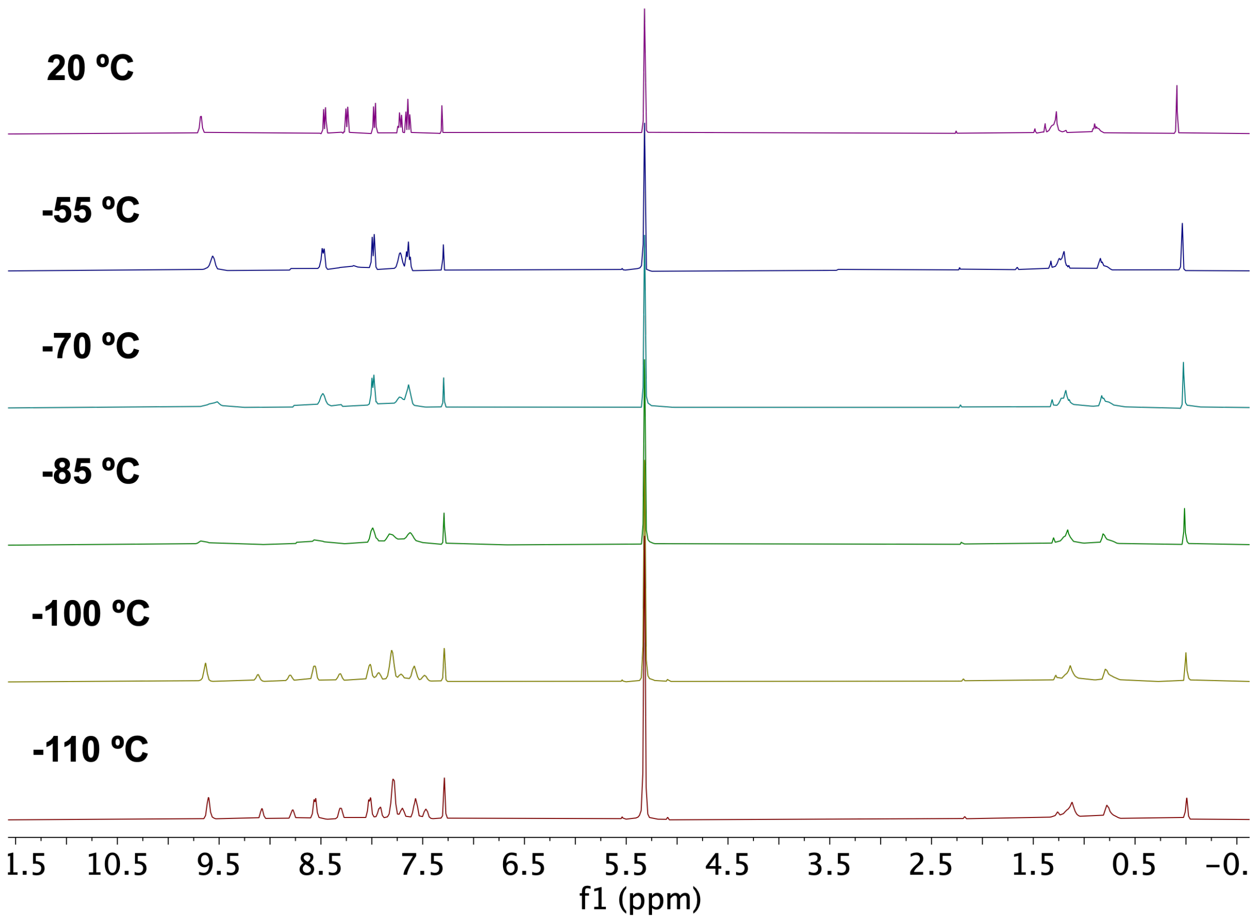

Figure S10. ^{1}H NMR spectra of {Q_3Sb(*o*-chlor)}Cu(OTf) (**3**) (60:27:13% by volume mixture of CD_2Cl_2:CCl_4:$CDCl_3$, 400 MHz) at various temperatures. Note: trace pentanes and silica grease impurities are present in the upfield region.

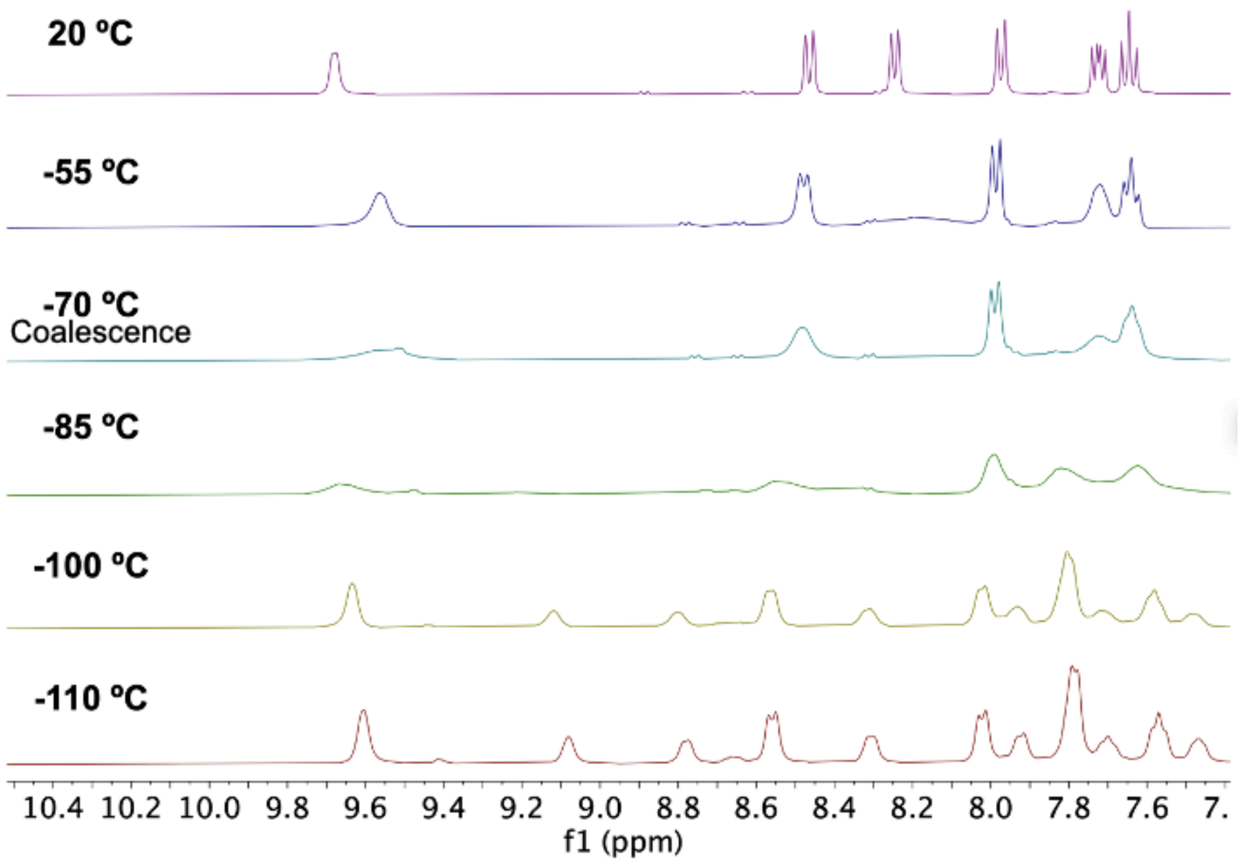

Figure S11. ^{1}H NMR spectra (aromatic region) of {Q_3Sb(*o*-chlor)}Cu(OTf) (**3**) (60:27:13% by volume mixture of CD_2Cl_2:CCl_4:$CDCl_3$, 400 MHz) at various temperatures. *Note*: trace intractable impurity is present in aromatic region.

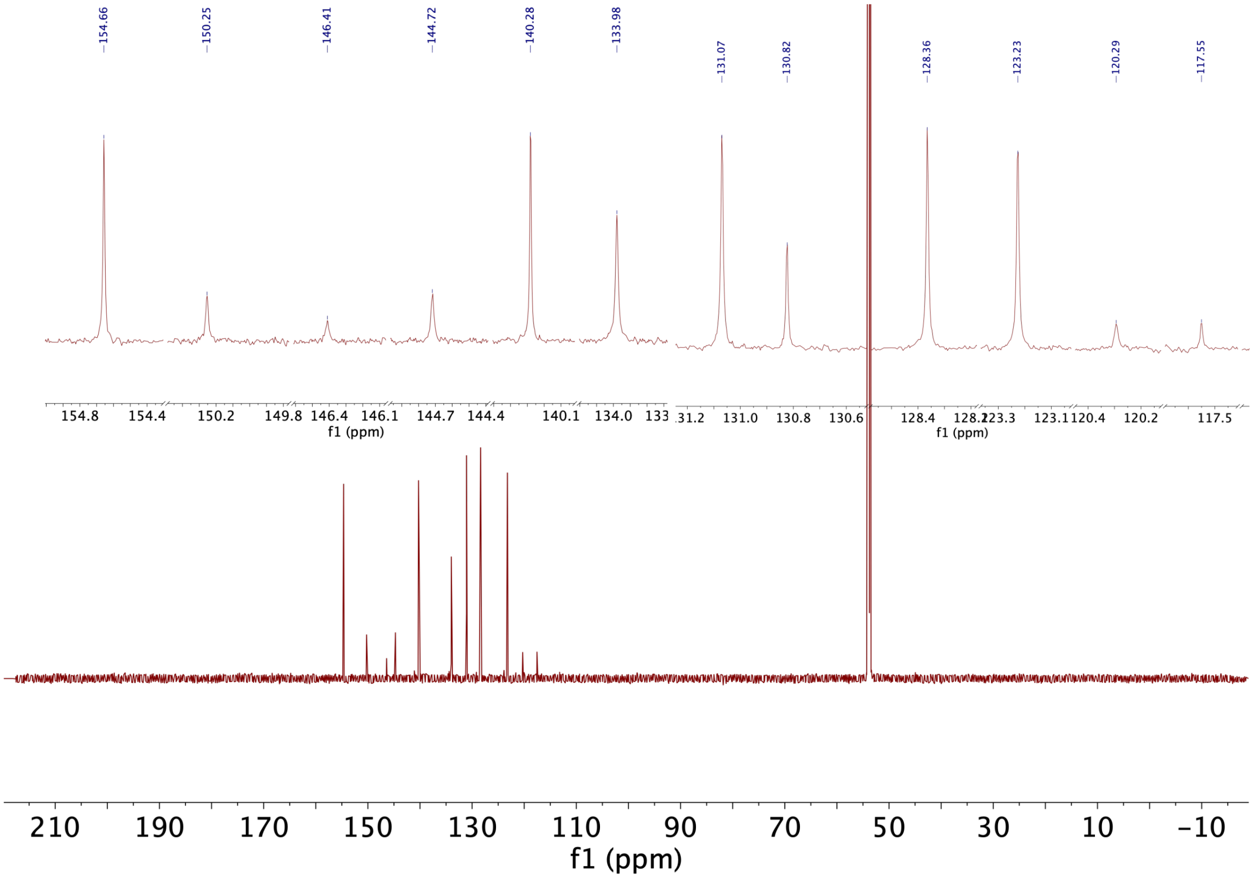

Figure S12. $^{13}C\{^{1}H\}$ NMR spectrum of $\{Q_3Sb(o\text{-chlor})\}Cu(OTf)$ (**3**) (201 MHz, CD_2Cl_2).

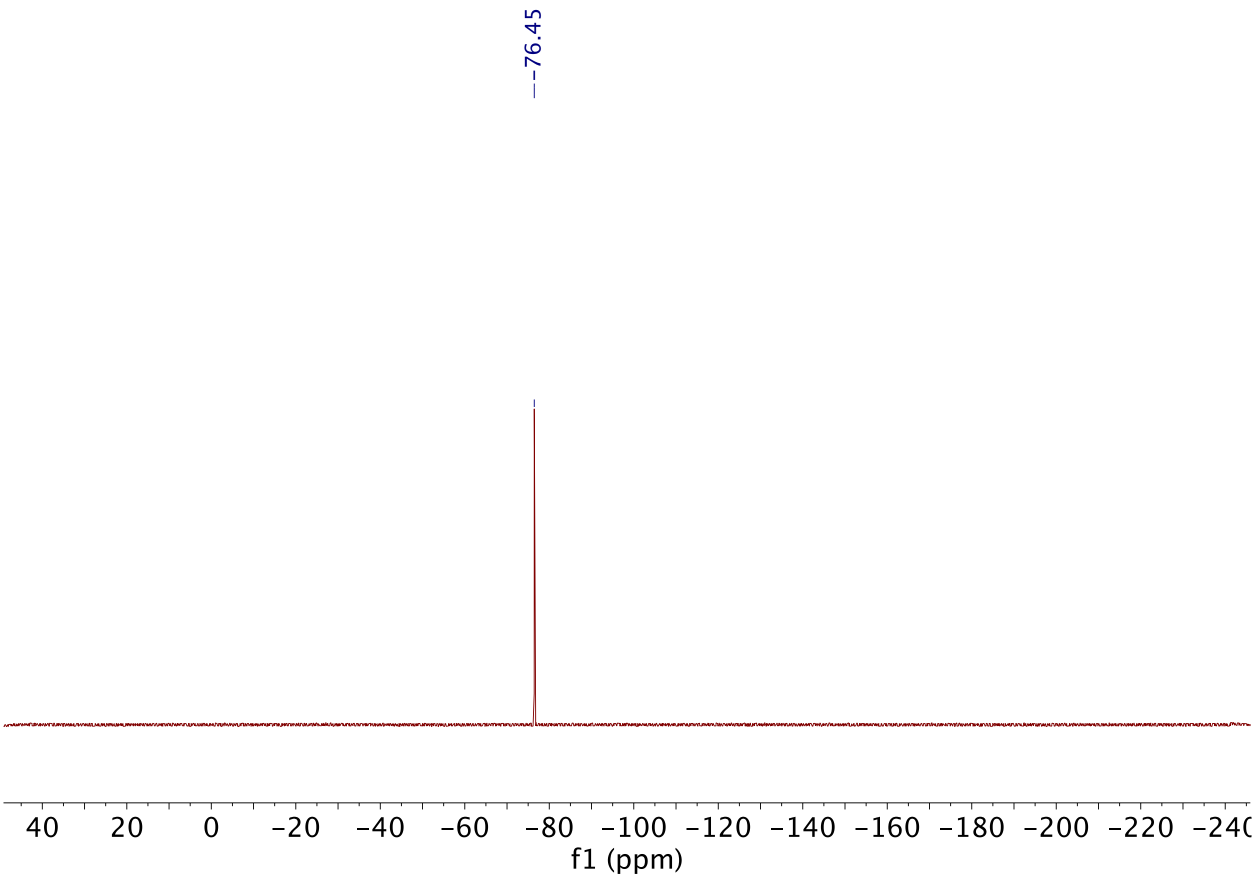

Figure S13. $^{19}F\{^1H\}$ NMR spectrum of $\{Q_3Sb(\textit{o}\text{-chlor})\}Cu(OTf)$ (**3**) (565 MHz, $CDCl_3$).

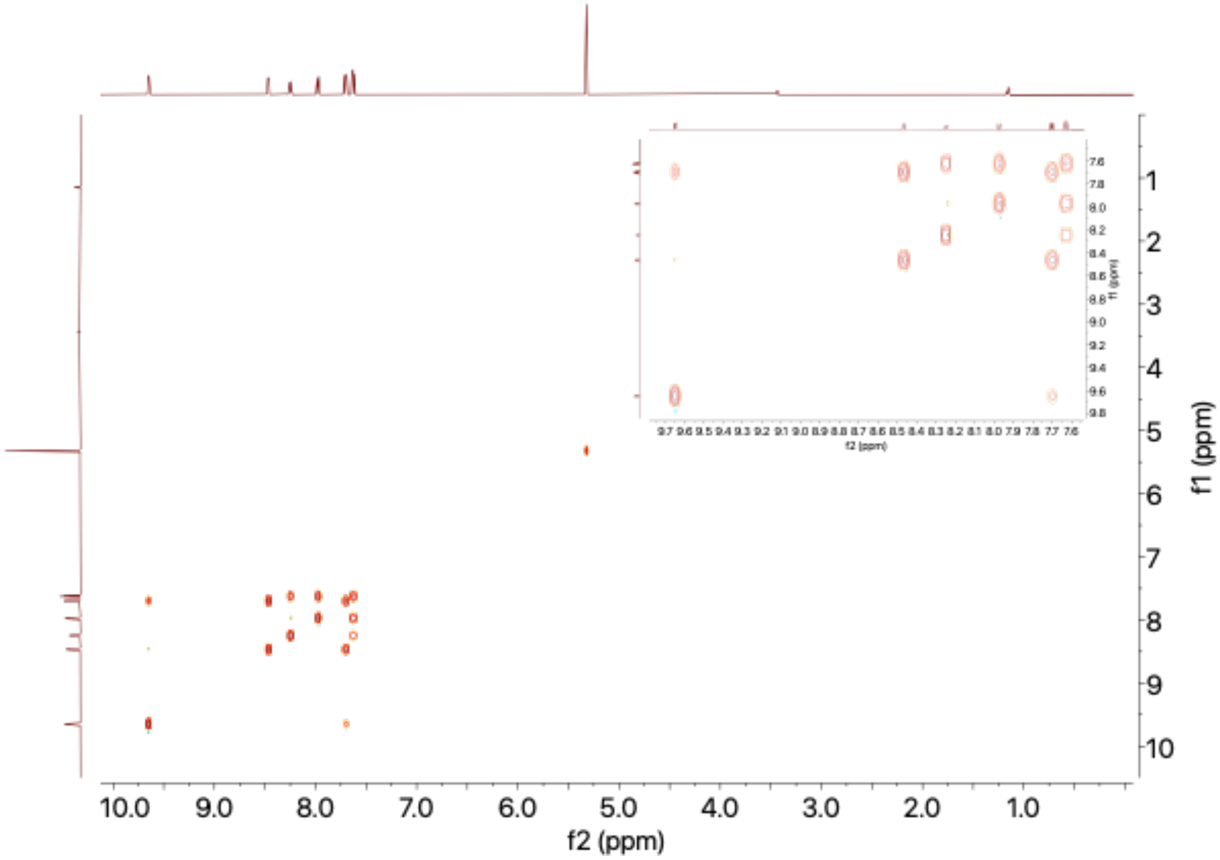

Figure S14. COSY NMR spectrum of {Q_3Sb(*o*-chlor)}Cu(OTf) (**3**) (400 MHz, CD_2Cl_2).

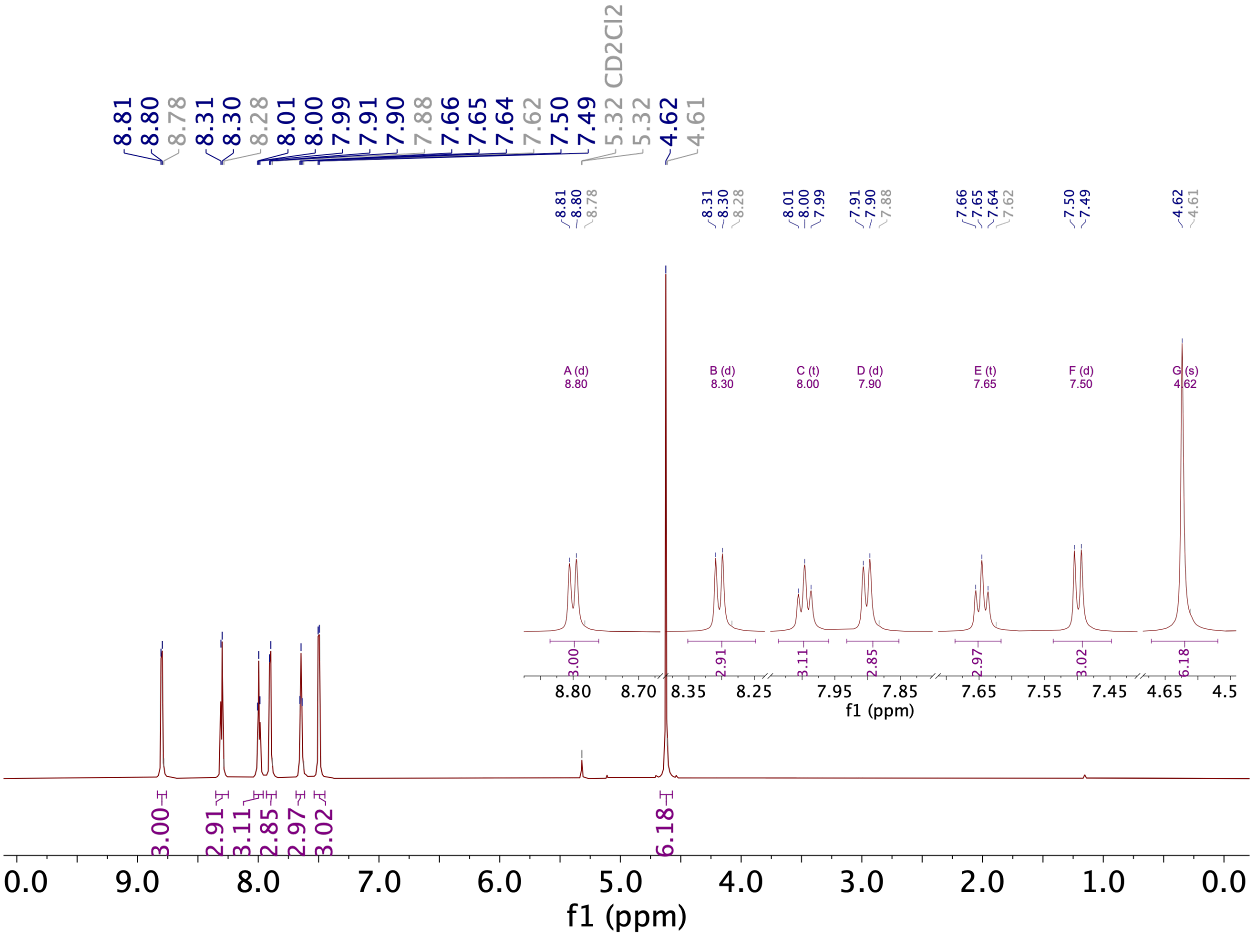

Figure S15. ^{1}H NMR spectrum of (TMQA)Cu(OTf) (**5**) (800 MHz, CD_2Cl_2).

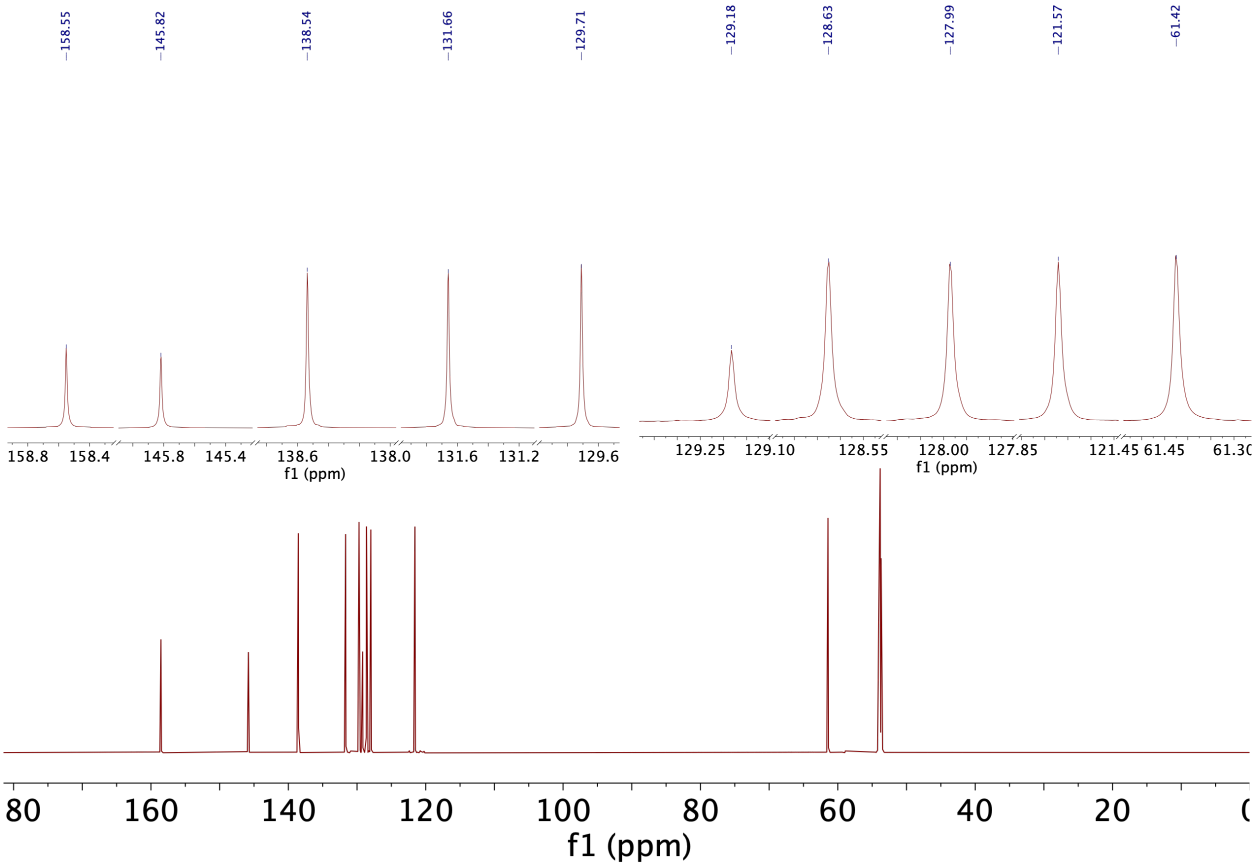

Figure S16. $^{13}C\{^1H\}$ NMR spectrum of (TMQA)Cu(OTf) (**5**) (201 MHz, CD_2Cl_2).

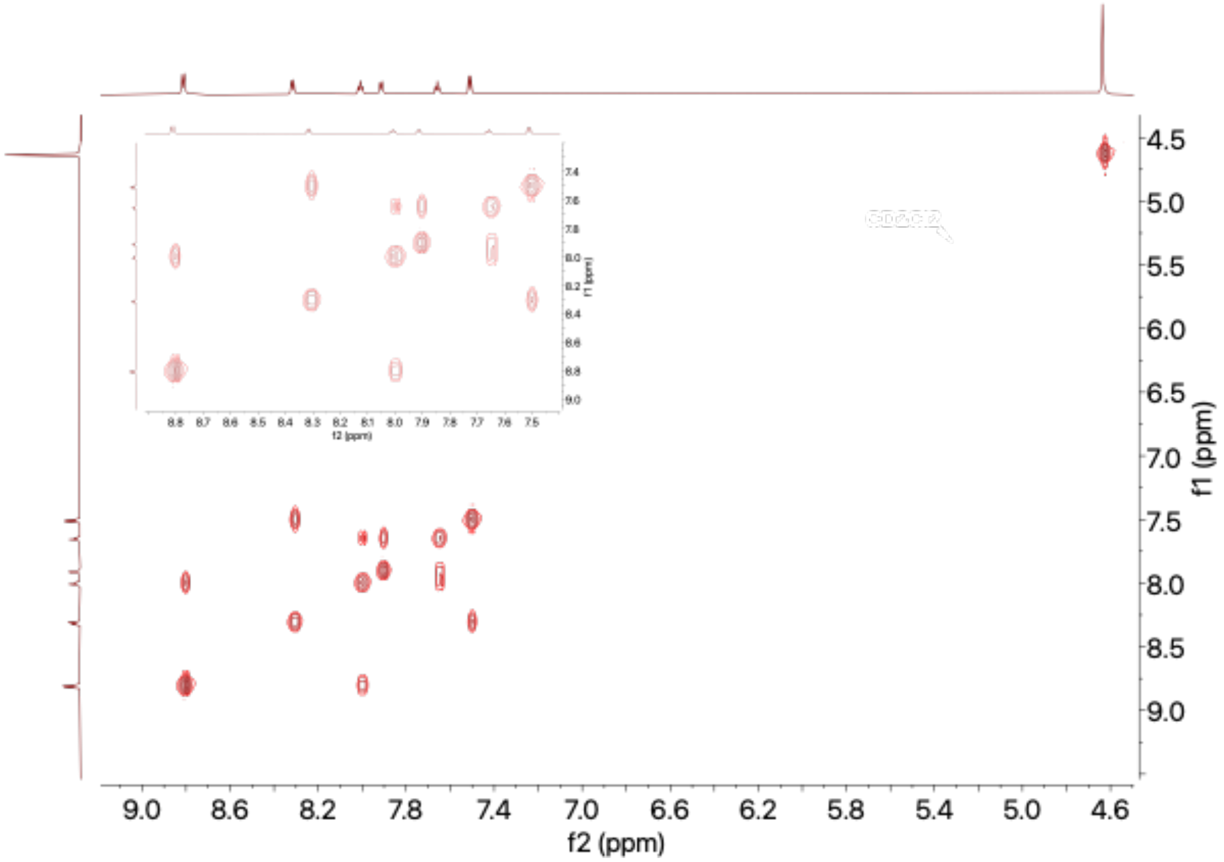

Figure S17. COSY NMR spectrum of (TMQA)Cu(OTf) (**5**) (800 MHz, CD_2Cl_2).

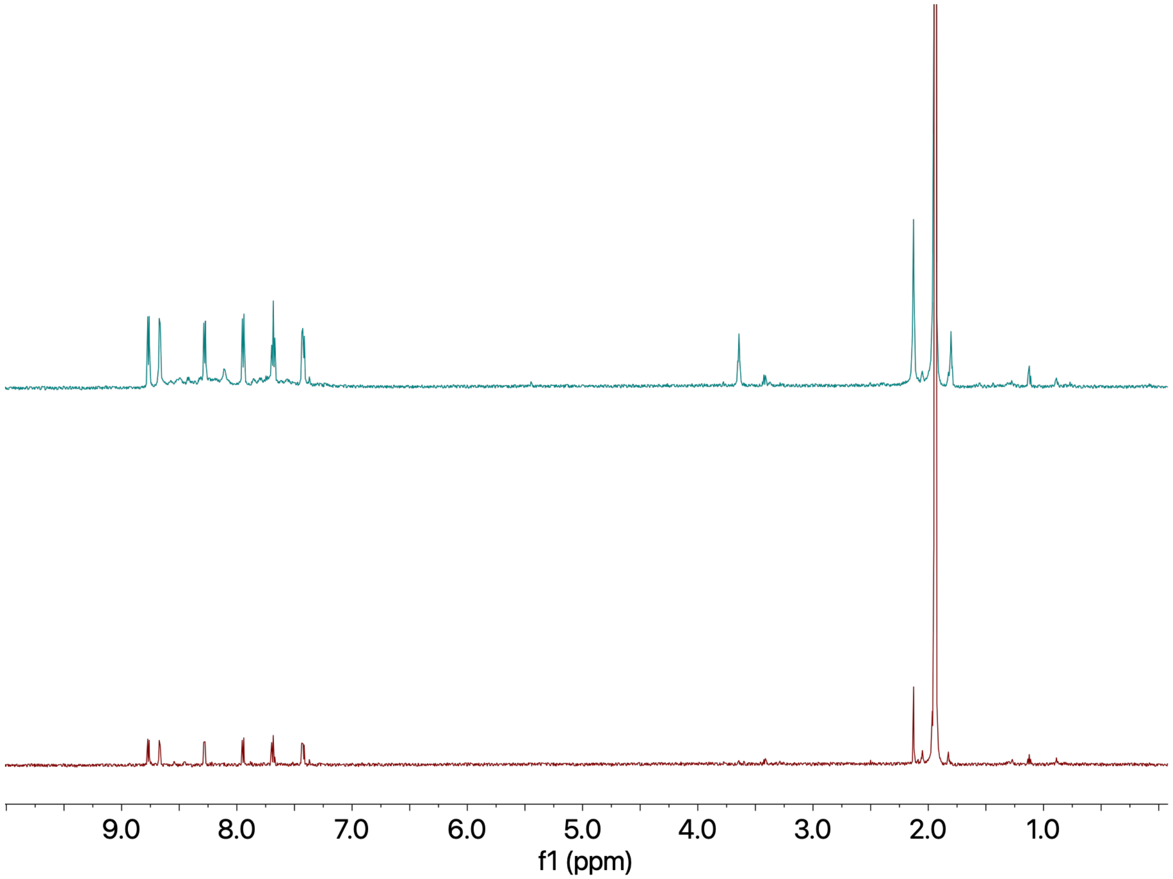

Figure S18. ^{1}H NMR spectra of Q_3Sb(o-chlor) (**2**, bottom) and {Q_3Sb(o-chlor)}Cu(OTf) (**3**, top) (600 MHz, d_3-MeCN). *Note:* Trace water and THF present in spectra.

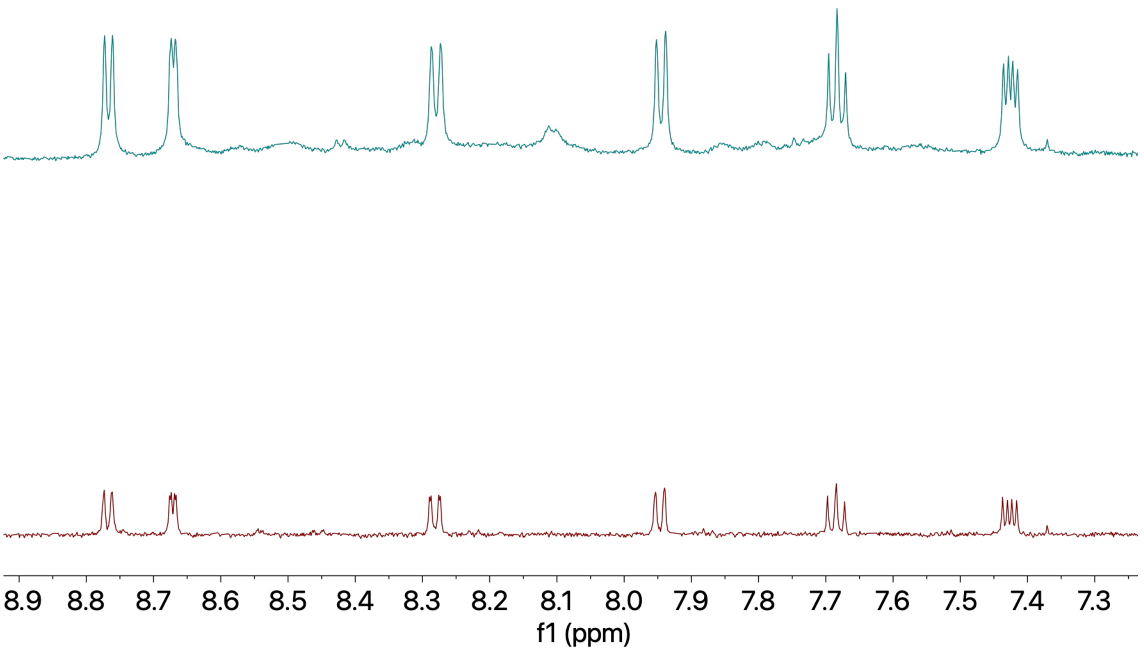

Figure S19. ^{1}H NMR spectra (aromatic region) of Q_3Sb(o-chlor) (**2**, bottom) and {Q_3Sb(o-chlor)}Cu(OTf) (**3**, top) (600 MHz, *d*$_3$-MeCN).

2. Calculation of Activation Energy Barrier for {Q_3Sb(*o*-chlor)}Cu(OTf)

Although we were unable to reach the slow exchange regime for {Q_3Sb(*o*-chlor)}Cu(OTf) (**3**), we implemented previously established methods to calculate $\Delta G^{\ddagger}$ at the lowest temperature to provide an upper limit of activation energy barriers. Following procedures first published by Shanan-Atidi and Bar-Eli,[1, 2] we calculated the ΔP using **Equation 1**. A value of 2 was used for P_{Major} since the mirror symmetric quinolines integrate for 2 protons and 1 was used for P_{Minor} leading to a value of 0.33 for ΔP.

Equation 1: $\Delta P = \frac{P_{Major} - P_{Minor}}{P_{Major} + P_{Minor}}$

We then used **Equation 2** to calculate X from ΔP. We obtained a value of X = 2.0869.[3]

Equation 2: $\Delta P = [\frac{X^2-2}{3}]^{\frac{3}{2}} \bullet \frac{1}{X}$

The free energies of activation for each proton environment can be calculated using a derivation of the Eyring Equation shown in **Equation 3**. Using the coalescence temperature (203 K) and the separation ($\partial\nu$) between the most downfield resonances at 163 K (208.86 Hz), we calculated an activation barrier upper limit of $\Delta G^{\ddagger} \leq 7.5$ kcal/mol.

Equation 3: $\Delta G^{\ddagger} = RTln[\frac{k_B}{h\pi}\left(\frac{T_C}{\partial_\upsilon}\right)\left(\frac{X}{1+\Delta P}\right)]$

3. Cyclic Voltammetry Studies

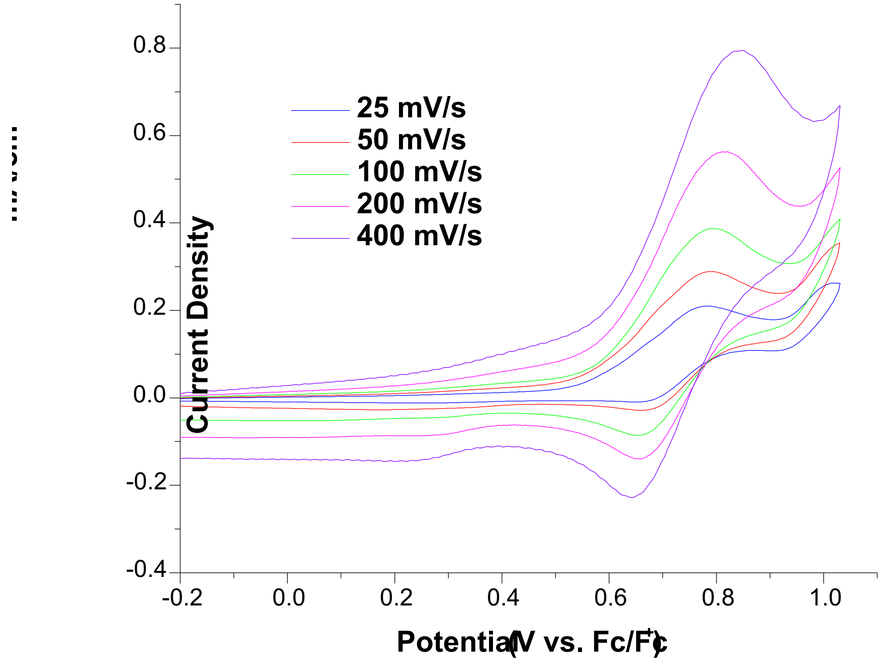

Figure S20. Scan rate study of {Q_3Sb(*o*-chlor)}Cu(OTf) (**3**) in DCM using a glassy carbon working electrode, platinum counter wire, $TBAPF_6$ supporting electrolyte with ferrocene as the internal reference added at the end of the experiment.

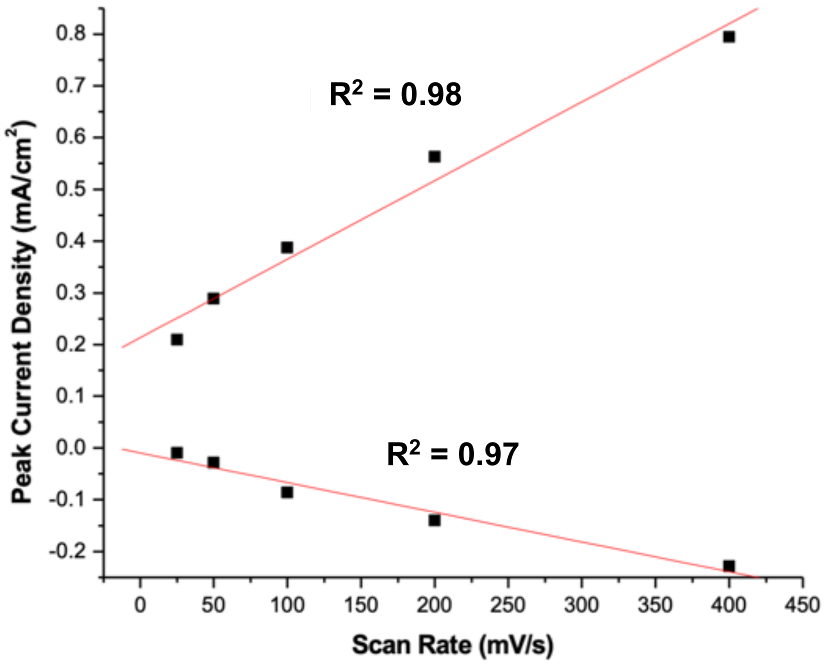

Figure S21. Plot of peak current density (mA/cm^2) vs. scan rate (mV/s) for {Q_3Sb(*o*-chlor)}Cu(OTf) (**3**) fitted linearly.

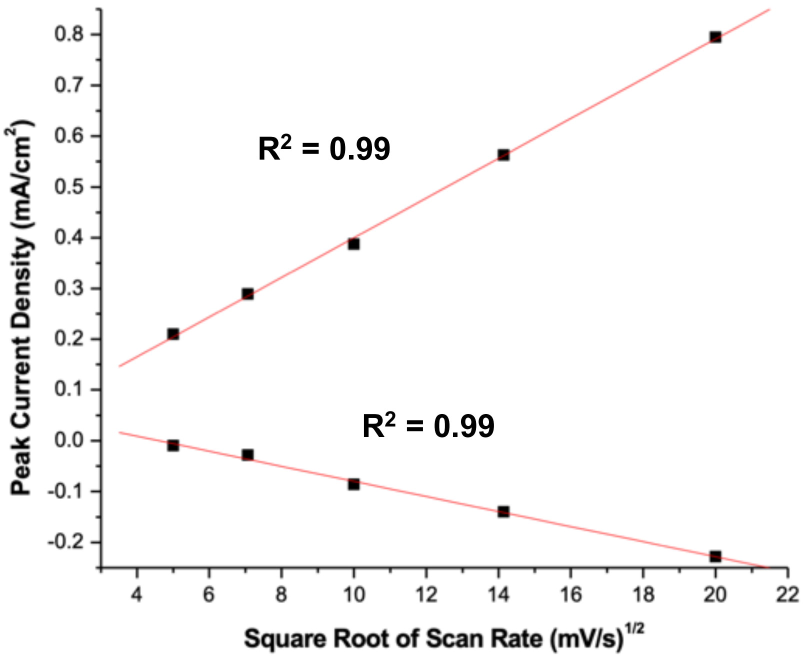

Figure S22. Plots of peak current density (mA/cm^2) vs. square root of scan rate $(mV/s)^{1/2}$ of $\{Q_3Sb(\textit{o}\text{-chlor})\}Cu(OTf)$ (**3**) with linear fits.

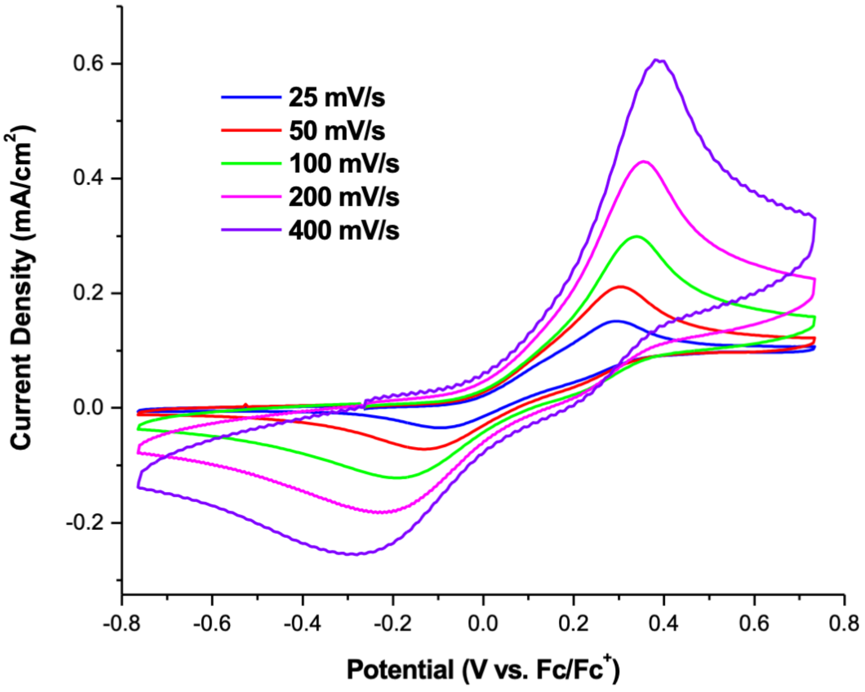

Figure S23. Scan rate study of (TMQA)Cu(OTf) (**5**) in DCM using a glassy carbon working electrode, platinum counter wire, $TBAPF_6$ supporting electrolyte with ferrocene as the internal reference added at the end of the experiment.

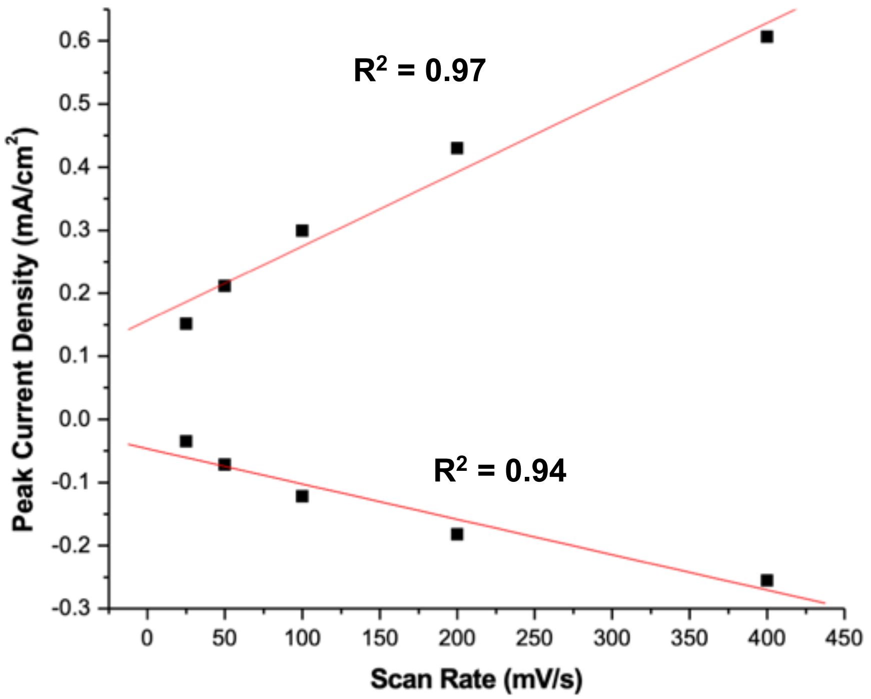

Figure S24. Plots of peak current density (mA/cm^2) vs. scan rate (mV/s) for (TMQA)Cu(OTf) (**5**) with linear fits.

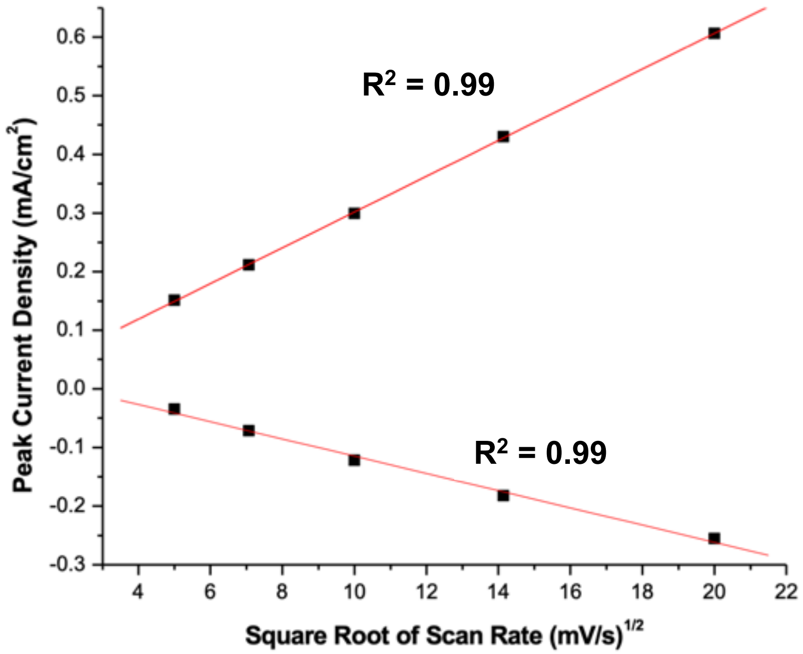

Figure S25. Plots of peak current density (mA/cm^2) vs. scan rate (mV/s) for (TMQA)Cu(OTf) (**5**) with linear fits.

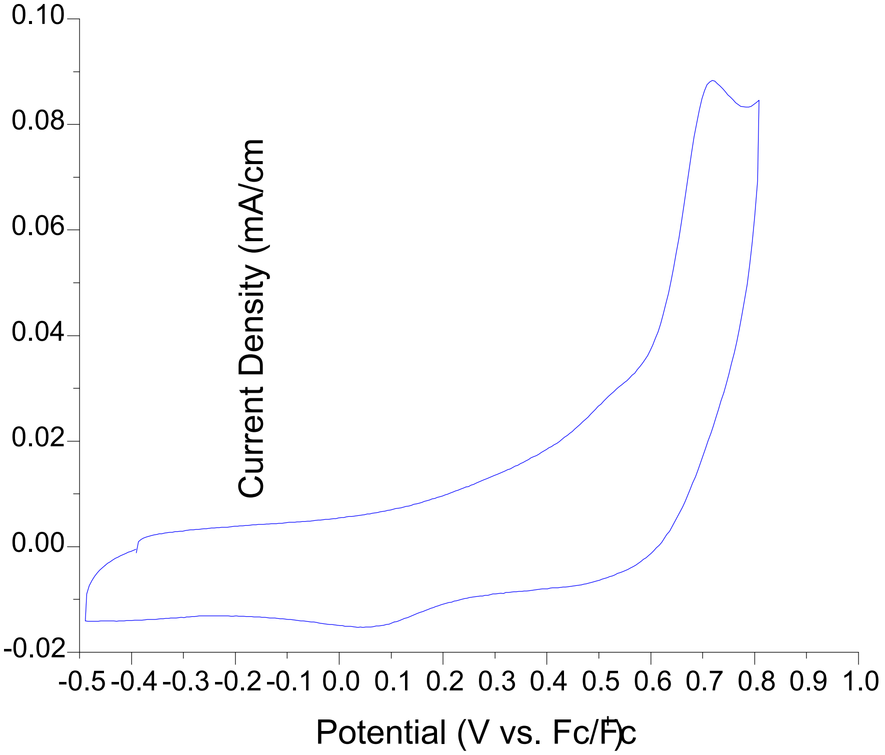

Figure S26. Cyclic voltammogram of {Q_3Sb(*o*-chlor)}Cu(OTf) (**3**) in propylene carbonate (0.8 mM due to low solubility) (PC) (0.8 mM [**3**] due to low solubility of **3** in PC) using a glassy carbon working electrode, platinum counter wire, $TBAPF_6$ supporting electrolyte with ferrocene as the internal reference added at the end of the experiment.

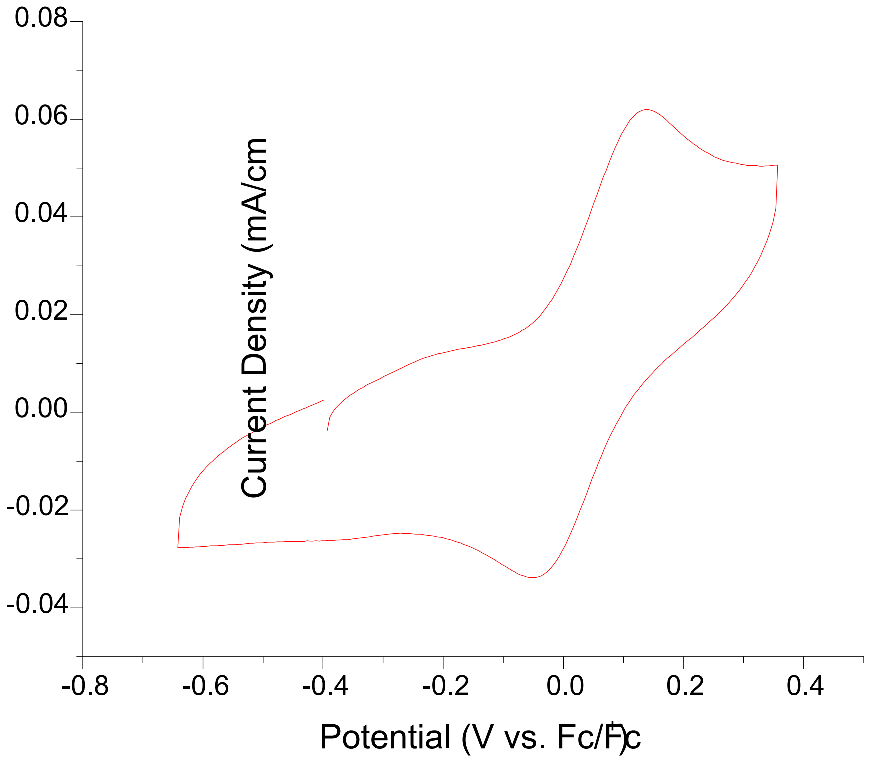

Figure S27. Cyclic voltammogram of (TMQA)Cu(OTf) (**5**) in propylene carbonate (0.8 mM due to low solubility) (PC) (0.8 mM [**5**] for consistent comparison with **3**) using a glassy carbon working electrode, platinum counter wire, $TBAPF_6$ supporting electrolyte with ferrocene as the internal reference added at the end of the experiment.

4. Crystallographic Data

Single crystals of Q_3Sb(*o*-chlor) (**2**), {Q_3Sb(*o*-chlor)}Cu(OTf) (**3**) and (TMQA)Cu(OTf) (**5**) were coated with Paratone oil and mounted on a MiTeGen MicroLoop. The X-ray intensity data of **5** were measured on a Bruker D8 Venture Photon III Kappa four-circle diffractometer system equipped with an Incoatec IμS 3.0 micro-focus sealed X-ray tube (Mo K_α, λ = 0.71073 Å for **2** and **5** and Cu K_α, λ = 1.54178 Å for **3**) and a HELIOS double bounce multilayer mirror monochromator. The frames were integrated with the Bruker SAINT software package[4] using a narrow-frame algorithm. Data were corrected for absorption effects using the Multi-Scan method (SADABS).[4] The structures were solved and refined using the Bruker SHELXTL Software Package[5] within APEX5[4] and OLEX2.[6]

All the non-hydrogen atoms were refined anisotropically. Hydrogen atoms were placed in geometrically calculated positions with $U_{iso} = 1.2U_{equiv}$ of the parent atom.

Table S1. Crystal structure data for Q_3Sb(*o*-chlor) (**2**), {Q_3Sb(o-chlor)}Cu(OTf) (**3**) and (TMQA)Cu(OTf) (**5**).

	**2**	**3**	**5**
CCDC number	2466395	2429426	2429427
Empirical formula	$C_{34}H_{20}Cl_6N_3O_2Sb$	$C_{69}H_{38}Cl_{10}Cu_2F_6N_6O_{10}S_2Sb_2$	$C_{31}H_{24}CuF_3N_4O_3S$
Formula weight	836.98	2014.25	653.14
Temperature [K]	100	100	100
Wavelength [Å]	0.71073	1.54178	0.71073
Crystal size [mm^3]	0.032×0.069×0.299	0.050 x 0.058 x 0.096	0.132× 0.506 × 0.718
Crystal habit	colorless plate	yellow needle	orange plate
Crystal system	monoclinic	monoclinic	triclinic
Space group	P 2_1/c	P 2_1/c	P-1
a [Å]	17.4424(8)	9.0105(2)	12.1887(7)
b [Å]	18.9803(8)	21.8982(5)	13.3541(7)
c [Å]	9.8250(4)	17.9983(5)	17.2652(11)
α [°]	90	90	81.277(2)
β [°]	96.437(2)	90.705(2)	83.870(2)
γ [°]	90	90	84.094(2)
Volume [$Å^3$]	3232.2(2)	3551.04(15)	2751.1(3)
Z	4	2	4
ρ_{calc} [gcm^{-3}]	1.720	1.884	1.577
μ [mm^{-1}]	1.388	11.353	0.933
F(000)	1656	1980	1336
θ range [°]	2.35 to 28.32	3.18 to 68.33	2.39 to 25.71
Index ranges	−23 ≤ h ≤ 23<br>−24 ≤ k ≤ 25<br>−13 ≤ l ≤ 10	-10 ≤ h ≤ 10<br>-26 ≤ k ≤ 26<br>-21 ≤ l ≤ 21	-14 ≤ h ≤ 14<br>-16 ≤ k ≤ 16<br>-21 ≤ l ≤ 21
Reflections collected	42264	33798	74220
Independent reflections	8016<br>[R_{int} = 0.0606]	6494<br>[R_{int} = 0.1198]	10456<br>[R_{int} = 0.1070]
Data / Restraints / Parameters	8016 / 0 / 415	6494 / 0 / 496	10456/0/775
Goodness-of-fit on F^2	1.021	1.020	1.023
Final *R* indexes [$I \geq 2\sigma(I)$]	R_1 = 0.0418<br>wR_2 = 0.1038	R_1 = 0.0496<br>wR_2 = 0.1070	R_1 = 0.0789<br>wR_2 = 0.2022
Final *R* indexes [all data]	R_1 = 0.0632<br>wR_2 = 0.1153	R_1 = 0.0840<br>wR_2 = 0.1238	R_1 = 0.1176<br>wR_2 = 0.2366
Largest peak/hole [$eÅ^{-3}$]	1.34/−0.94	0.936/-0.870	1.237/-0.498

5. Details of XPS Experiments

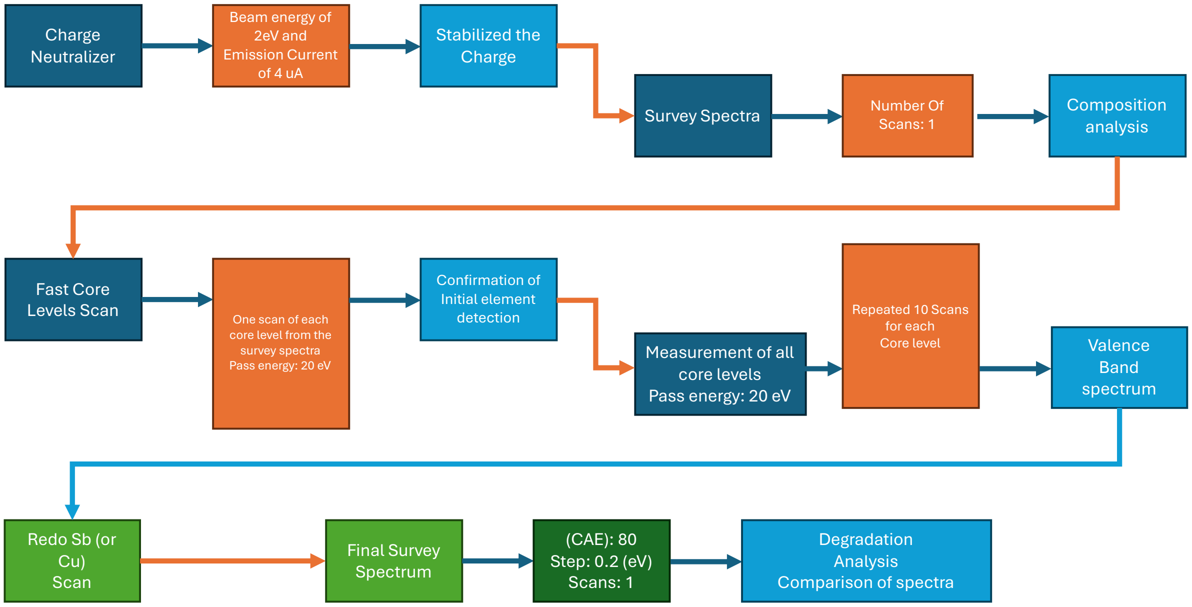

Figure S28. Representative workflow used to assess radiation damage during the XPS measurements.

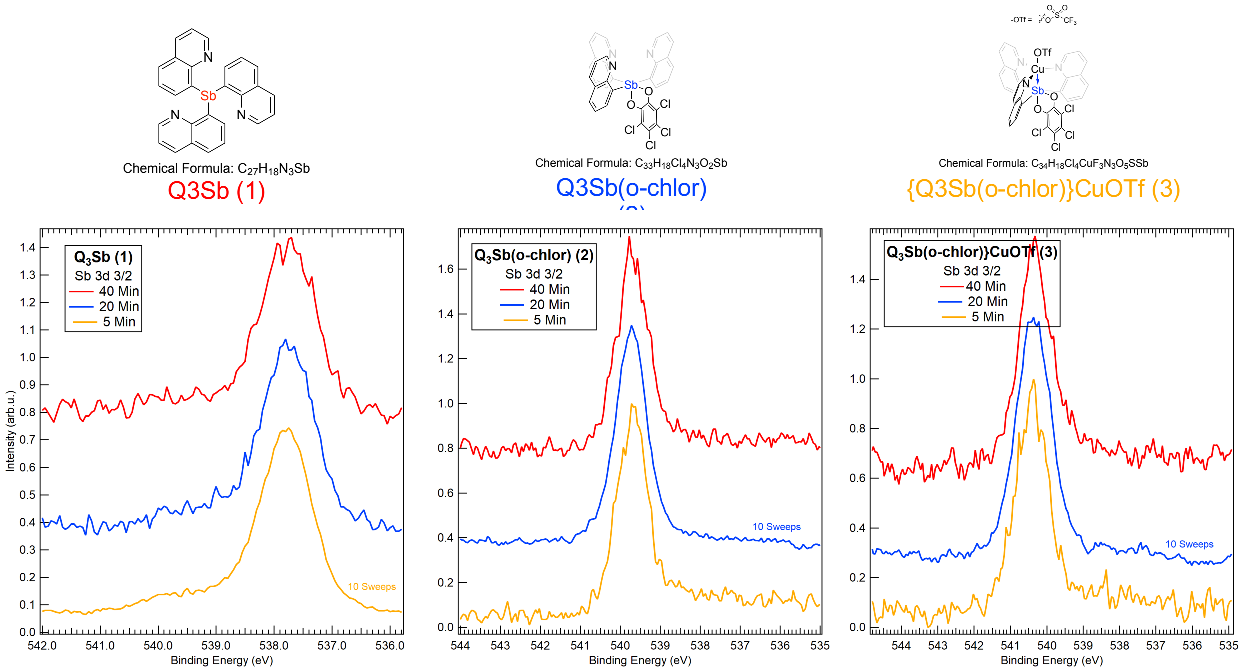

Figure S29. Examples of Sb 3d core level spectra recorded at various times throughout the degradation assessment. Note that for M1 10 scans were accumulated after 5 min, while for all others 10 scans were recorded for a total of 20 min. Apart from the change in the signal-to-noise ratio due to variation in the number of scans, the spectra are identical.

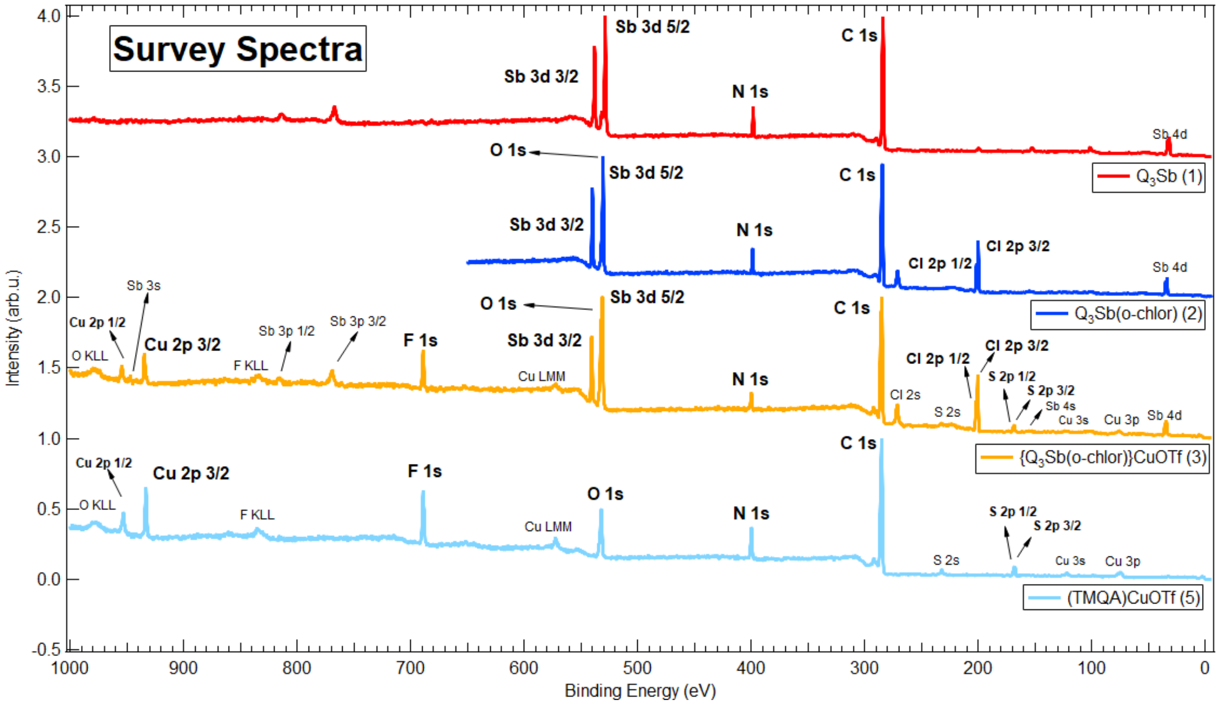

Figure S30. Survey spectra for all molecules studied with XPS. All core levels and Auger peaks are labeled. For M1 minute contamination with Cl, and Cu are seen < 200 eV.

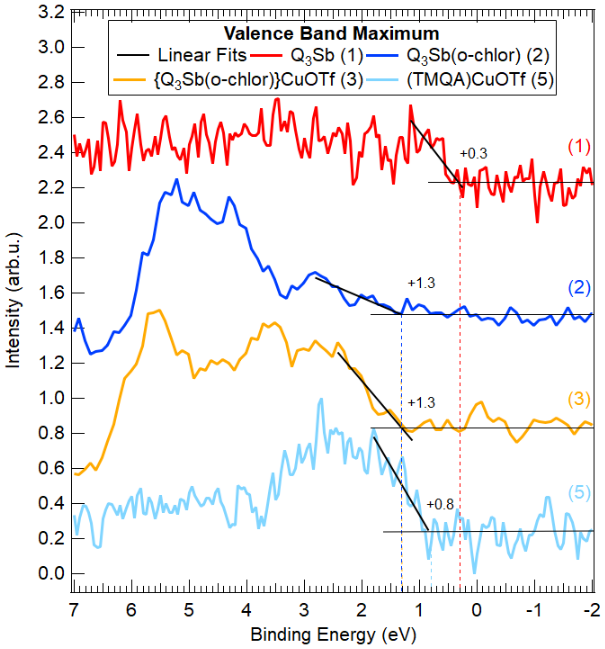

Sample	VBM	Shift for Alignment
Q_3Sb (1)	0.3	-0.3±0.1
Q_3Sb(o-chlor) (2)	1.3	-1.3±0.2
{Q_3Sb(o-chlor)}CuOTf (3)	1.3	-1.3±0.2
(TMQA)CuOTf (5)	0.8	-0.8±0.1

Figure S31. Determination of the valence band maximum (VBM) position with respect to the Fermi level. The error is from the measurement and extrapolation of the leading edge of the spectrum to the baseline. All spectra are shifted so the valence band is positioned at the origin of the binding energy scale E(VBM) = 0.

Table S2. Molecule composition from XPS measurement in at% and comparison with nominal molecule composition. The error from measurement, integration, and fit is about ±2% for at% values. The higher Oxygen percentage indicates the presence of adsorbates and the molecules might be hygroscopic. The element contributions are otherwise accurate within the margin of error.

O 1s and Cl2p contributions to (1) are contamination and adsorbates. C1s peaks can also include some adventitious carbon and as such is overrepresented in the composition values measured with XPS.

		Sb 3d	Cl 2p	N 1s	C 1s	O 1s	F 1s	Cu 2p	S 2p
Complex (1)	(1) XPS at%	4.9%	1.6%	7.8%	82.7%	3.0%			
	XPS Composition	1	0	2	17	1			
	Composition at%	3.2%	0.0%	9.7%	87.1%	0.0%			
	Composition	1	0	3	27	0			
Complex (2)	(2) XPS at%	4.3%	18.4%	8.7%	52.0%	16.6%			
	XPS Composition	1	4	2	12	4			
	Composition at%	2.3%	9.3%	7.0%	76.7%	4.7%			
	Composition	1	4	3	33	2			
Complex (3)	(3) XPS at%	3.4%	16.8%	6.8%	40.7%	21.5%	6.2%	1.6%	3.0%
	XPS Composition	1	6	2	14	7	2	1	1
	Composition at%	1.9%	7.7%	5.8%	65.4%	9.6%	5.8%	1.9%	1.9%
	Composition	1	4	3	34	5	3	1	1
Complex (5)	(5) XPS at%			14.9%	43.8%	19.3%	12.0%	3.5%	6.2%
	XPS Composition			2	7	3	2	1	1
	Composition at%			9.3%	72.1%	7.0%	7.0%	2.3%	2.3%
	Composition			4	31	3	3	1	1

Table S3. Core level positions for all elements with respect to the Fermi level and the valence band maximum (VBM). The error in position is +/- 0.05 eV for all molecules with respect to the Fermi level. The error in position with respect to the valence band maximum (VBM): (**1**) +/- 0.1 eV, (**2**) +/-0.2 eV, (**3**) +/- 0.2 eV, (**5**) +/-0.1 eV. These values are also tabulated in.

Core level positions are given in units of eV.		Q_3Sb (1)	Q_3Sb(o-chlor) (2)	{Q_3Sb(o-chlor)}CuOTf (3)	(TMQA)CuOTf (5)
Sb $3d_{3/2}$	Position – E_F	537.8	539.7	540.4	
	Position-VBM	537.5	538.4	539.1	
Cl $2p_{5/2}$	Position – E_F	198.2	200.0	200.3	
	Position-VBM	197.9	198.7	199.0	
N 1s	Position – E_F	398.1	398.7	399.8	399.7
	Position-VBM	397.8	397.4	398.5	398.9
C 1s	Position – E_F	283.6	284.3	284.8	284.8
	Position-VBM	283.3	283.0	283.5	284.0
O 1s	Position – E_F	530.4	531.2	532.1	531.9
	Position-VBM	530.1	529.9	530.8	531.1
F 1s	Position – E_F			688.7	688.5
	Position-VBM			687.4	687.7
Cu $2p_{5/2}$	Position – E_F			934.1	932.8
	Position-VBM			932.8	932.0
S $2p_{5/2}$	Position – E_F			168.1	167.8
	Position-VBM			166.8	167.0

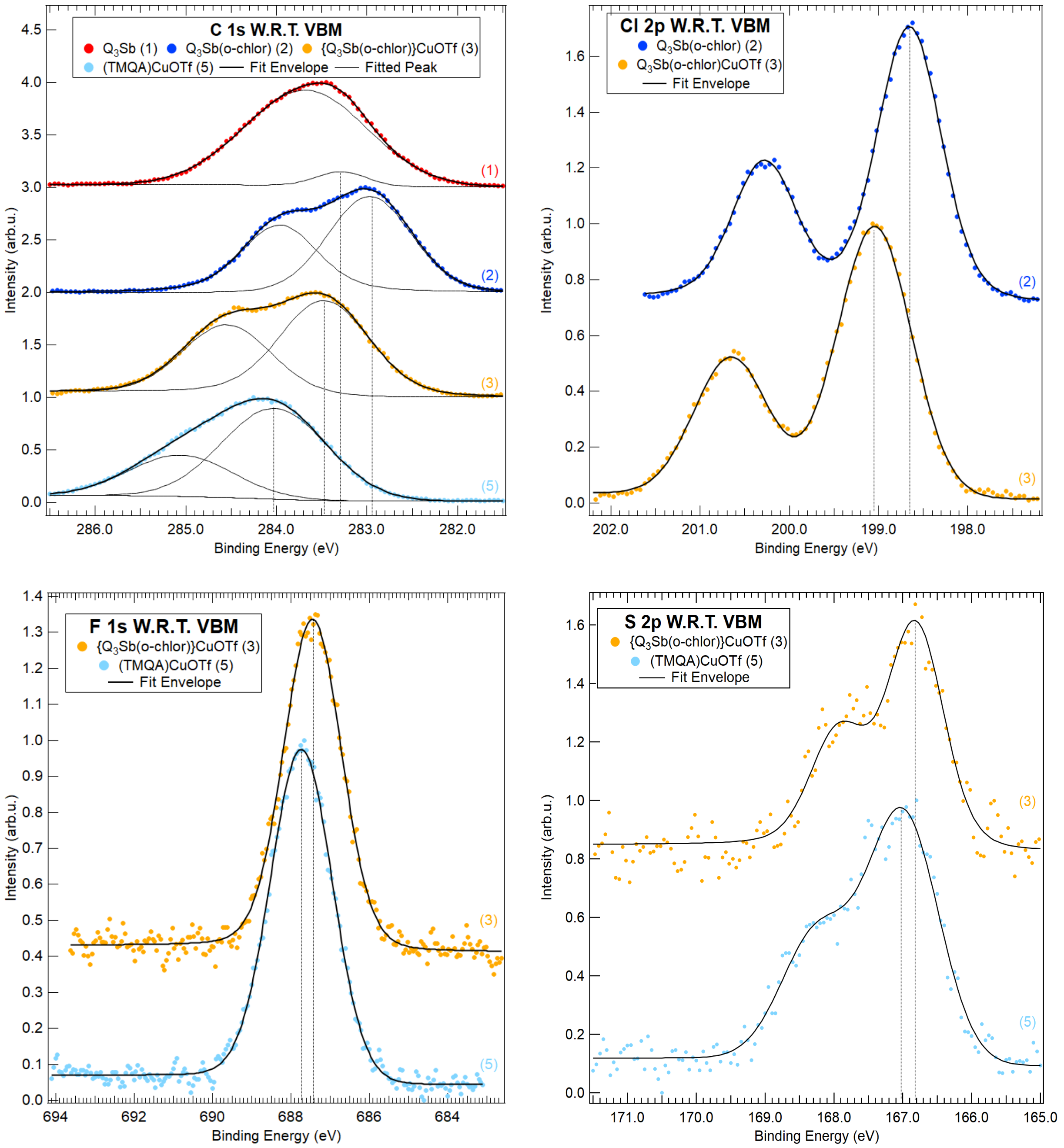

Figure S32. Core levels for C 1s, Cl 2p, F 1s and S 2p for all four molecules as applicable. All core levels are displayed with the binding energy referenced to the VBM.

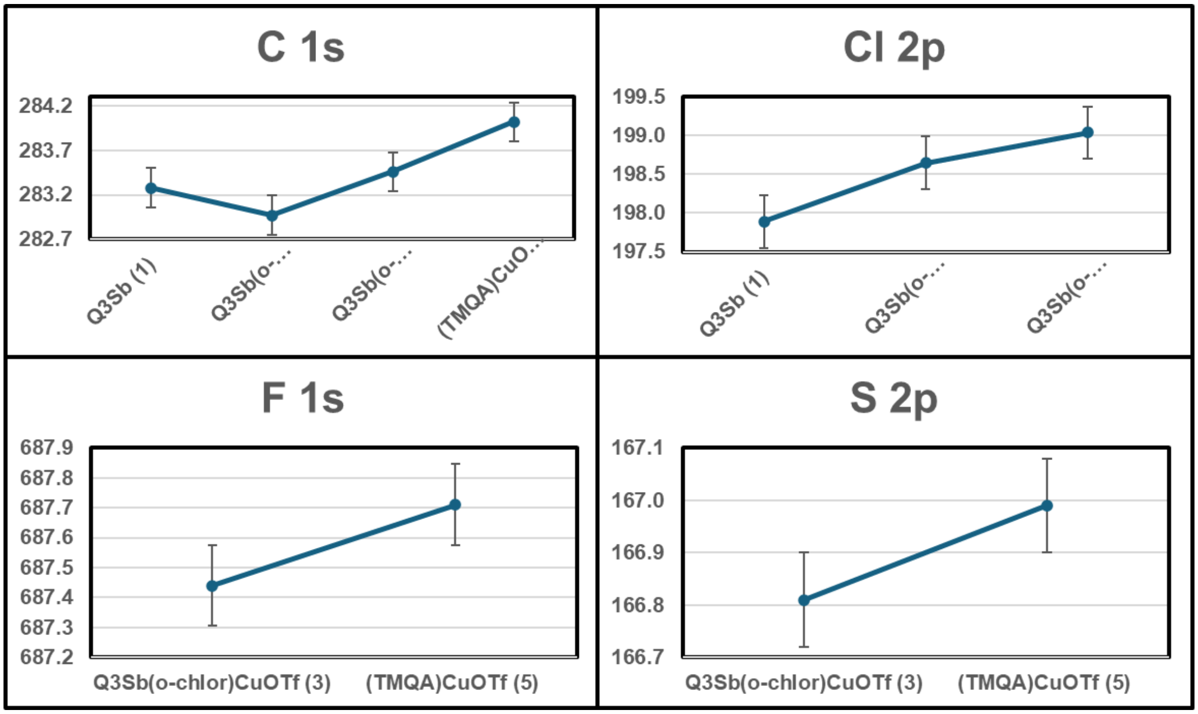

Figure S33. Core levels for C 1s, Cl 2p, F 1s and S 2p core levels for all four molecules as applicable. All core levels are displayed with the binding energy referenced to the VBM. Note Cu, Sb, N1s and O1s are included in the main body of manuscript figure.

7. References

(1) Shanan-Atidi, H.; Bar-Eli, K. H. A convenient method for obtaining free energies of activation by the coalescence temperature of an unequal doublet. *J. Phys. Chem.* **1970**, *74* (4), 961-963. DOI: 10.1021/j100699a054.

(2) Oldham, W. J.; Heinekey, D. M. Synthesis of Ethylene Hydridotris(1-pyrazolyl)borate Triphenylphosphine Complexes of Rhodium and Iridium and Their Reactions with Hydrogen. *Organometallics* **1997**, *16* (3), 467-474. DOI: 10.1021/om9605140.

(3) Rösch, A. T.; Söntjens, S. H. M.; Robben, J.; Palmans, A. R. A.; Schnitzer, T. Rotational Isomerism of an Amide Substituted Squaraine Dye: A Combined Spectroscopic and Computational Study. *The J.Org. Chem.* **2021**, *86* (18), 13100-13103. DOI: 10.1021/acs.joc.1c00922.

(4) *Saint; SADABS; APEX3.*; Bruker AXS Inc.: Madison, Wisconsin, USA., 2012.

(5) Sheldrick, G. M. SHELXT - integrated space-group and crystal-structure determination. *Acta Cryst. Sect. A Found Adv.* **2015**, *71* 3-8. DOI: 10.1107/s2053273314026370

(6) Dolomanov, O.; Bourhis, L.; Gildea, R.; Howard, J.; Puschmann, H. OLEX2: A complete structure solution, refinement and analysis program. *J. Appl. Cryst.* **2009**, *42*, 339-341. DOI: 10.1107/S0021889808042726.
